# Unveiling the Effects of Thermal Aging on the Oxidative Stability of Biobased Low-Density Polyethylenes

**DOI:** 10.1021/acsomega.5c00636

**Published:** 2025-06-11

**Authors:** Joanna Aniśko-Michalak, Anatolij Sokolohorskyj, Izabela Szafraniak-Wiza, Paulina Kosmela, Adam Piasecki, Mateusz Barczewski

**Affiliations:** † Faculty of Mechanical Engineering, Institute of Materials Technology, Polymer Processing Division, 49632 Poznan University of Technology, Piotrowo 3, Poznań 61-138, Poland; ‡ Department of Polymers, University of Chemistry and Technology, Prague, Technická 5, Prague 6 166 28, Czech Republic; § Faculty of Materials Engineering and Technical Physics, Institute of Materials Engineering, Poznan University of Technology, Al. Jana Pawła II 24, Poznań 61-138, Poland; ∥ Department of Polymer Technology, Gdansk University of Technology, Narutowicza 11/12, Gdańsk 80-233, Poland

## Abstract

The study evaluates biobased polyethylene (LDPE) structural changes induced by long-term thermal oxidation. The three grades of LDPE differentiated with structural properties were exposed to 90, 100, and 110 °C in an air atmosphere for 20 days. After aging, samples were comprehensively analyzed by Fourier transform infrared (FTIR) spectroscopy, focused on carbonyl group development, and quantified by the carbonyl index (CI). Detailed analysis, based on the deconvolution of the FTIR spectra, identifies each carbonyl compound, whose content varies depending on the structure of the LDPE. The changes in the crystallinity were investigated via X-ray diffraction (XRD) and differential scanning calorimetry (DSC). The obtained results were supplemented by oxidation induction time (OIT), oxygen onset temperature (OOT), and an in-depth analysis of the rheological properties. The tested polymers undergo degradation with behavior similar to UV degradation of petrochemical ones, and the cross-linking process surpasses chain scission for samples degraded at 100 and 110 °C.

## Graphical abstract

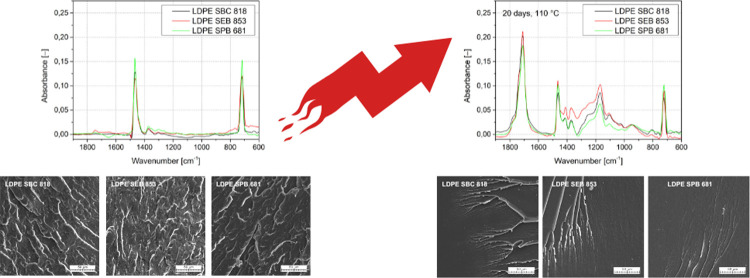

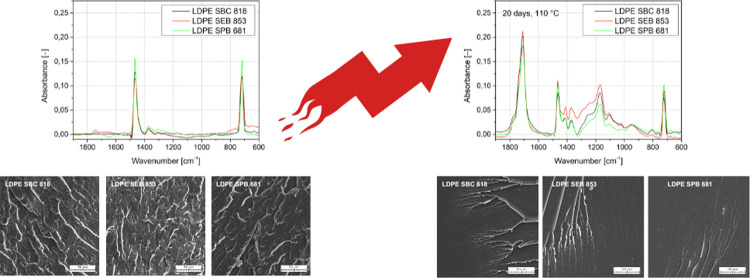

## 1 Introduction

Polyethylene (PE) is one of the most used polymers, which can be found in household items, plastic bags, bottles, pipes, and others. The global production of polyethylene in 2021 was 107 million metric tons,^1^ which caused considerable environmental damage. The production of plastics from fossil fuels causes greenhouse gas emissions (GHGs); for example, plastic production in the USA is responsible for 1% of total greenhouse gas emissions. One of the promising alternatives to reduce these environmentally unfavorable phenomena is the introduction of polyethylene from renewable resources. Analysis of life cycle greenhouse gas emission confirms that the GHG emission for fossil-derived PE is higher than for biobased PE processed the same way. In this way, the CO_2_ emission can be reduced from 2.6 and 2.9 kg of CO_2_ per kg of fossil-derived HDPE and LDPE to −1.0 kg of CO_2_ per kg of biobased PE.^2^ The main reason biobased polyethylene has lower GHG emissions is because of biomass carbon uptake—another reason the biobased PE development is related to the decreasing amount of fossil fuels.^3^ Nowadays, the production of plastics should be less dependent on fossil fuel resources. As previously mentioned, polyethylene is used to produce everyday products and many single-use plastic goods. The politics from the European Commission’s European Green Deal of 2020 prepared the Single-Use Plastics Directive, which defines the framework for biobased and biodegradable polymers. This directive assumes the withdrawal from the market of disposable and single-use products like straws, cups for beverages, plates, and cutlery and an increase in recycling.^4^

Biobased polyethylene is the most promising replacement for fossil-derived PE. The synthesis routes of BioPE differ from those of fossil-based PE.^5^ Biopolyethylene synthesis is based on the polymerization of ethylene monomer by dehydration of bioethanol from glucose. Glucose can be obtained from several biological feedstocks like sugar cane, sugar beet, starch crops, wheat, and lignocellulosic materials.^3,6^ The biobased polyethylene can be named ″green polymers″ because of another great advantage of this material: the sugar cane plant, which they are made of, can capture CO_2_ from the atmosphere. According to Braskem, 2.5 tons of CO_2_ are captured by sugar cane plants for each ton of green polyethylene produced. This will allow for the maintenance of CO_2_ balance after the combustion of green PE.^5,7^ On the other hand, the petrochemical low-density polyethylene is produced via free-radical polymerization of ethylene monomer at high temperature and pressure.^8,9^ The main difference is the source of the ethylene monomer. In conventional polyethylene, this monomer is obtained as a petroleum distillation product via thermal cracking of hydrocarbons at high temperatures (750–1000 °C).^10^

Biobased polyethylene is supposed to have the same properties as its petrochemical counterpart since its chemical structure is the same, but there is a lack of investigation of its thermal aging behavior. Polyolefins are sensitive to degradation at high temperatures. The presence of oxygen accelerates these effects and causes the oxidation of material and deterioration of its structure and properties.^11^ The thermal decomposition of polyethylene starts with cleavage of the C–C bond. Subsequently, the rest of the thermal reaction mechanisms occur, like scission, H-abstraction, intramolecular hydrogen (backbiting) transfer, and intermolecular hydrogen shift (random scission), which lead to the monomer yield from the polymer.^12^ This reaction forms chain radicals that react with oxygen to form peroxide radicals.^13,14^ There are two primary mechanisms in this process: initiation: RH → R^•^ + H^•^, where the chain radicals are formed, and the second is propagation R^•^ + O_2_ → ROO^•^, and then the peroxy radicals are formed. After these steps, the intermolecular and intramolecular hydrogen abstraction reaction occurs. They lead to the obtaining of new radicals and, therefore, accelerate the oxidation mechanism. Below are presented schemes of the formation of radicals in the polyethylene oxidation process

I $${ROO}^{•}+RH\to ROOH+R^{\cdot }$$

II $$ROOH\to {RO}^{•}+OH$$

III $$ROOH+RH\to {RO}^{•}+R^{•}+HOH$$

IV $$RH+{RO}^{•}\to ROH+R^{•}$$

Throughout the reaction (I), hydroperoxide is formed, and then the breakdown of hydroperoxide occurs (II).^15^ The secondary alkoxyl radical (RO^•^) can abstract the H atom from this decomposition to form alcohol or cause the backbone scission by the β-scission reaction (5).^16^ This reaction can propagate finally to the termination of the polymer chains.
V

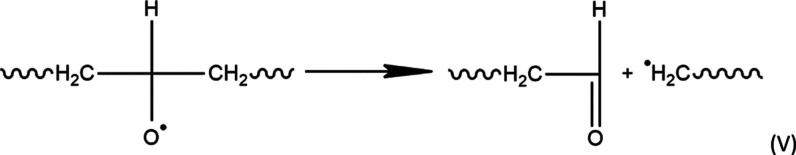




Other courses of the reactions that propagate further the polyethylene oxidation, according to Gugumus,^17^ are presented below

VI $${RO}^{•}+RH\to ROH+R^{•}$$

VII $${RO}^{•}\to {\left(R=O\right)}_{aldehyde}+R^{•}$$

$$R^{•}+O_{2}\to {{RO}_{2}}^{•}$$ VIII

IX $${ROO}^{•}+RH\to ROOH+R^{•}$$

X $${ROO}^{•}+RH\to {\left(ROOH\right)}_{a}+R^{•}$$

XI $${ROO}^{•}+RH\to R=O+R^{•}$$

XII ROOH•→R=O+OH•

Reaction (IX) is the usual way of presenting this reaction. Reaction (X) is shown as an alternative hydroperoxide formation method, and the same reaction can rely on the direct formation of carbonyl/carboxyl groups. Then, the next step in the oxidation of polyethylene is the termination, where compounds without radicals are formed.^17^

XIII $${ROO}^{•}\to R=O+ROH$$

XIV $${ROO}^{•}+{ROO}^{•}\to R=O+ROH$$

XV $${ROO}^{•}+R^{•}\to ROOR$$

XVI $${2R}^{•}\to R-R$$

The termination step results in obtaining a neutral product by eliminating two stable radicals to not initiate chain breaking again.^18^ In the reactions (XIII–XVI), the termination possibilities are presented. Usually, the polymer chain undergoes scission reactions during the thermal degradation of polyethylene, but cross-linking is another significant degradation mechanism.^19^ The cross-linking occurs less frequently than the chain scission mechanism, but it still can be observed for LDPE samples aged at 80 °C.^20^ Cross linking occurs through two reactions: the junctions of the vinylidene group with a skeleton alkyl, leading to linkages and formation of two tertiary carbons separated by a methylene group and the reaction of secondary alkyls to form a covalent bond between tertiary carbon atoms.^19^ The reactions that lead to that mechanism are the same as those for the oxidation mechanism, where the initiation and formation of radicals is followed by the formation of the alkoxyl radical and the β-scission reaction.^21^ There are many options for polyethylene’s thermal degradation, which depend on temperature, time, and even heating rates.

Growing interest in biopolymers raises concerns about their processability and thermal stability. The thermo-oxidative stability of polymers derived from petrochemicals and biobased sources depends on several material characteristics, such as molecular weight, chemical structure, oxygen penetration, etc. For example, thermal oxidation of starch-based biopolymers is challenging due to their complex multicomponent chemical structures; radicals from different constituents can interact, forming various degradation products and influencing the degradation rate.^22^ Another group of popular biobased polymers is biopolyesters, e.g., poly­(lactic acid), which undergo degradation during processing due to temperature, oxygen, and mechanical stress.^23^ The complex degradation phenomena consist of thermal and thermo-oxidative degradation, which occurs at the processing temperature (∼200 °C) and follows a random chain scission mechanism.^24^ More recent developments consider the production of nonbiodegradable polymers from biobased resources, like polyethylene from ethylene monomer of plant origin. Since the production of biopolyethylene has a limited negative impact on the environment compared to petrochemical grades, the main goal is to increase the use of this material in manufacturing consumer products. This study will clarify misconceptions about using sustainable resources in polyethylene synthesis, which could substantially impact the advancement of nonbiodegradable thermoplastics derived from biological sources. This work is carried out as experimental screening research investigating the thermal oxidation of biobased polyethylene according to the knowledge described in the literature. The mechanisms of these reactions discussed in this paper concern three commercially available grades of biobased low-density polyethylene, varying in dispersity.

## 2 Materials

### 2.1 Materials and Preparation

Three grades of biobased low-density polyethylene (LDPE) were processed to investigate their properties after thermal oxidation. Materials were obtained from Braskem (Brazil), LDPE SBC 818 I’m Green, LDPE SEB 853 I’m Green, LDPE SPB 681 I’m Green. The densities of these three biopolyethylenes provided by the manufacturer were 0.918 g/cm^3^, 0.923 g/cm^3^, and 0.922 g/cm^3^, respectively. The melt flow indexes for these bioLDPE materials in the conditions 190 °C/2.16 kg are 8.3 g/10 min (SBC 818), 2.7 g/10 min (SEB 853), and 3.8 g/10 min (SPB 681). According to the material data sheets provided by the producer and conducted research in this study, polyethylene grades are nonstabilized and additive-free.

To obtain samples for the thermal oxidation process, biopolyethylene materials were dried at 50 °C for 24 h in a vacuum dryer and then compression-molded into samples with a 25 mm diameter and a 1 mm thickness using a laboratory hydraulic press, Remiplast (Poland). The compression molding process was carried out for 2 min at 160 °C and 18 MPa; then, samples were taken out from between heated plates, placed under load, and cooled using forced air flow for 5 min.

### 2.2 Thermal Oxidation

Compression-molded disk-shaped samples were thermally oxidized using a laboratory dryer Memmert ULE 500 with forced airflow. Samples were placed in aluminum pans in driers at elevated temperatures of 90 °C, 100 °C, and 110 °C. The thermal oxidation step process took 20 days, and samples were removed from the driers after 1, 2, 5, 8, 12, 16, and 20 days.

## 3 Methods

Molecular weights of polyethylenes were determined using a high-temperature GPC-IR instrument (Polymer Char) equipped with an infrared and viscometric detector. Separation was carried out on two Olexis mixed columns (Polymer Laboratories, 13 μm) at 150 °C in 1,2,4-trichlorobenzene at an elution rate of 1 mL·min^–1^. Molecular weights were calculated using the universal calibration approach in the GPC One software.

A Jasco FT/IR-4600 Fourier transform infrared (FTIR) spectrometer was used to characterize the structural changes. The FTIR spectra were obtained by 32 scans at a resolution of 4 cm^–1^ in the wavenumber range of 4000–400 cm^–1^. Data obtained from this test was used to calculate the carbonyl index (CI) using eq 1, which allows a quantitative definition of the rate of oxidation.

1 $$CI=\left(\frac{A_{\left(C=O\right)}}{A_{0}}\right)$$

In eq 1, A_(C=O)_ is the absorbance of the carbonyl group peak (1800–1650 cm-^1^); A_0_ is the absorbance band, which was selected as an internal reference due to its minimal band affection by the thermal oxidation.^25^ This band is related to methylene (CH_2_) from 1500 to 1420 cm^–1^.^26^ The carbonyl band was also deconvoluted to investigate which carbonyl compounds are produced at different stages of thermal oxidation. The second derivative method in the OriginPro software was used to deconvolute the carbonyl band. The curve fitting was also carried out in OriginPro software using the Gaussian profile, and the quality of the obtained fitting was controlled by the coefficient of determination (*R* ^2^ > 0.99). The FTIR measurement was performed again to do this evaluation, but in the wavenumber range of 1800–1650 cm^–1^ by 64 scans, the scanning resolution was 4 cm^–1^. The data from curve fitting was also used to measure the carbonyl compound concentration according to the Beer–Lambert law (2).^27^

2 $$C=\frac{IA}{\varepsilon \cdot b}$$

where *C* is the molar concentration [mol/L], *IA* is the integrated absorbance (area), *b* is the thickness of the sample, which is in the considered case 0.1 cm, and ε is the molar extinction coefficient [L·mol^–1^·cm^–1^]. For the ketone compounds, the molar extinction coefficients are 300 L·mol^–1^·cm^–1^, 680 L·mol^–1^·cm^–1^ for acids, 450 L·mol^–1^·cm^–1^ for esters, and 720 L·mol^–1^·cm^–1^ for lactones.^28,29^

The crystallographic structure of the materials was analyzed by X-ray diffraction (XRD) with Cu K_α_ radiation (λ = 1.54 Å) using the Panalytical Empyrean model (Almelo, The Netherlands). The conditions of the XRD measurements were as follows: voltage 45 kV, anode current 40 mA, 2θ range from 5° to 40°, time per step 60.214 s, step size 0.0165°. This evaluation helped to calculate the degree of crystallinity (*X*_c_) using eq 3

3 $$X_{c}=\frac{I_{c}}{I_{c}+I_{a}}\times 100\%$$

where *I*_c_ is the integrated area of the crystalline peak, and *I*_a_ is the integrated area of the amorphous phases. From this measurement, the crystallite size can be measured as well

4 $$P=\frac{k\lambda }{\beta \cos\theta }$$

where *k* is the Scherrer constant (0.9), λ is the wavelength of the X-ray radiation, and β is the half-width of the crystalline peak.^30^

Differential scanning calorimetry (DSC) measurements were realized using a NETZSCH DSC 204 F1 Phoenix apparatus with aluminum crucibles and 5 ± 0.1 mg samples under nitrogen flow. All samples were heated from 20 to 200 °C and held molten for 5 min. Then, the samples were cooled to 20 °C with a constant cooling rate of 10 °C/min. The temperature program was conducted twice to erase the samples’ thermal history. All of the studied materials remained thermally stable during the DSC measurements, as revealed by the additionally realized thermogravimetric analysis (TGA). To calculate the degree of crystallinity, the following equation was used

5 $$X_{C}=\frac{\Delta H_{m}}{\Delta H_{100\%}}\times 100\%$$

where Δ*H*_m_ is the melting enthalpy, and Δ*H*_100%_ is the melting enthalpy of 100% crystalline polyethylene.^31^

Differential scanning calorimetry (DSC) was applied to determine the oxidation induction time (OIT) for the polyethylene grades considered.^32^ For the purpose of the experiment, 5 ± 0.2 mg of specimens was used. They were heated from 20 to 190 °C (heating rate of 20 °C/min) under nitrogen flow and then kept at 190 °C for 5 min in nitrogen; the gas was then switched to oxygen, and the time required for sample oxidation was measured. When measuring the oxidation onset temperature (OOT), tests were conducted in an oxidizing atmosphere at a constant heating rate of 10 °C/min and ranged from 20 to 250 °C. The measurements were performed in a NETZSCH DSC 204 F1 Phoenix apparatus and aluminum crucibles.

Analysis of the rheological behavior of three grades of biobased polyethylene subjected to thermal aging was realized by oscillatory rheometry using an Anton Paar MCR 301 rotational rheometer. The measurements were conducted with 25 mm diameter parallel plates and a 0.5 mm gap. The experiments were conducted at 190 °C. The strain sweep experiments were conducted before the dynamic oscillatory measurements were performed in frequency sweep mode. The preliminary strain sweep experiments performed with a constant angular frequency of 6.28 rad/s in the varying strain window of 0.01–100% allow the determination of the linear viscoelastic range used for frequency sweep analyses. A 2% strain was applicable for frequency sweep experiments and was located in the linear viscoelastic (LVE) region for all samples. The frequency sweep measurements proceeded in the angular frequency range of 0.05–500 rad/s. The experimental oscillatory data for nontreated polyethylene grades showed shear thinning behavior and were fitted to the Carreau–Yasuda model described by the following formula

6 $$\eta \left(\mathrm{\gamma ̇}\right)=\eta_{0}{\left[1+{\left(\lambda \mathrm{\gamma ̇}\right)}^{a}\right]}^{n-1/a}$$

where η_0_ is the zero-shear viscosity, *n* is the power law coefficient, *a* is the parameter describing the width of the transition between the terminal and shear thinning regime, and λ is the characteristic relaxation time. The function parameters fitted to experimental data (Figure 12) are collectively presented in Table 4 with information about storage *(G*′*)* and loss moduli *(G″)* curves’ crossover point and calculated based on their relaxation time θ determined as 1/ω _*G*′=*G*″_.^33–35^

According to the International Commission on Illumination (CIE),^36^ sample colors were assessed using *Lab* coordinates. In this system, *L* represents color brightness (with *L* = 0 for black and *L* = 100 for white), *a* indicates the green(−)/red­(+) axis, and *b* signifies the blue(−)/yellow­(+) axis. Color assessment employed optical spectroscopy via the NR145 Precision Colorimeter 3nh Portable Spectrophotometer, housed within a specially designed light trap chamber with a white mated surface serving as the background. The yellowness index (YI) was calculated as described by eq 6 per the ASTM E313 standard, following the conversion of color parameters into CIE XYZ coordinates.^37^

7 $$YI=100\times \left(C_{X}X-C_{Z}Z\right)/Y$$

where *C*_X_ = 1.3013 and *C*_Z_ = 1.1498 for the D65 CIE standard illuminant and observer.

A scanning electron microscope, Tescan MIRA3 (Brno, Czech Republic), equipped with an EDS-UltimMax energy-dispersive spectrometer (Oxford Instruments, High Wycombe, UK) and Aztec Energy Live Standard software were used to assess the oxygen content in polyethylene aged samples’ cross-section. The samples were tested with an accelerating voltage of 12 kV and a working distance of 16 mm. Additionally, the cross-section of aged samples was analyzed at 1000× magnification to observe morphological changes in cryogenically fractured specimens.

## 4 Results and Discussion

### 4.1 Molecular Mass of Biobased Polyethylenes

Data presented in Figure S1 (Supporting Information) and Table 1 summarize the HT-GPC (high-temperature gel permeation chromatography) results for three raw and unprocessed LDPE grades as received in the form of pellets, including weight-average molecular mass (*M*_w_), dispersity (D̵), and molar mass at the maximum of the peak (Mp). The LDPE SBC 818 sample differs in the distribution of molecular weights and contains a fraction of chains with higher molecular mass. This bimodal distribution results in a broader dispersity compared to the other two samples, which exhibit a comparable distribution of molecular weights (LDPE SEB 853 and LDPE SPD 681). The difference in molar mass distribution may be related to its applicability, where polymers with higher molecular weights positively contribute to mechanical properties but simultaneously complicate processability. Processability significantly deteriorates with longer linear chains; hence, bimodal systems containing lower molecular weights are often preferred today. These systems help reduce the viscosity of the molten material. For longer PE chains, there is also a slightly higher probability of random scission, which is related to the increased number of breakable sites (individual C–C bonds)—the effect of longer chains is not so significant as branching along the chain.^38,39^ The weight-average molecular weights (*M*_w_) correlate with the melt flow indexes listed in the material data sheets. The LDPE SBC 818 sample, with the highest molecular weight (∼140 kg/mol), exhibits a significantly higher melt flow index (8.3 g/10 min) compared to the other two samples with a molecular weight of ∼80 kg/mol, which have MFIs of 2.7 g/10 min and 3.8 g/10 min, respectively. The differences in MFIs are also influenced by the macromolecular structures of the samples. The LDPE SBC 818 sample, due to its lower density (0.918 g/cm^3^), is expected to be more linear compared to LDPE SEB 853 and LDPE SPD 681 (0.923 g/cm^3^ and 0.922 g/cm^3^, respectively). Plots of normalized IR signal versus elution volume, used for calculating molecular weight according to the methodology described by Sun et al.,^40^ are additionally summarized in Figure S1 in the Supporting Information.

**Table 1:** Molecular Weight and Dispersity of LDPE Grades before Aging Determined by HT-SEC in 1,2,4-Trichlorobenzene at 150 °C

Sample	*M*_w_ (kg mol^–1^)	D̵ (−)	*M*_p_ (kg mol^–1^)	Molecular weight distribution mode
LDPE SBC 818	140.3	10.24	44.1	bimodal
LDPE SEB 853	80.7	6.16	61.7	unimodal
LDPE SPB 681	76.3	5.78	59.9	unimodal

### 4.2 Spectroscopic Analysis

The thermal degradation of biobased polyethylene grades was performed in an air-circulating oven at 90, 100, and 110 °C for a maximum time of 20 days. The chemical composition changes were investigated by FTIR-ATR spectroscopy. This method has some limitations due to the restricted depth of the penetration (*d*_p_) sample with IR radiation, which depends on the wavelength (λ), incident angle (θ), prism refractive index (*n*_1_), and sample refractive index versus prism (*n*_2,1_).^41^ All these factors are gathered into eq 7, specifying the penetration depth.^41–43^

8 $$d_{p}=\frac{\lambda }{2\lambda n_{1}\sqrt{{\left(\sin\theta \right)}^{2}-{\left(\frac{n_{1}}{n_{2}}\right)}^{2}}}$$

This penetration depth is usually around 2 μm^42^ or, as stated by Liu and Kazarian,^43^ in the 0.2–5 μm range. This characteristic of FTIR-ATR spectroscopy restricts the analysis of the changes in the chemical composition in the whole bulk of the samples; mainly, surface effects are observed. In this study, the 1 mm samples are subjected to thermal degradation; they are not applicable for the FTIR test in transmission mode; so to overcome this, the ATR mode is performed. Even though it has some drawbacks, it is frequently used to evaluate aged polymers.^26,44–46^ The studies focusing on the intensity of penetration of harmful oxygen and UV rays into the polymer matrix showed that changes in the carbonyl index at a depth of 1 mm are negligible for LDPE,^47^ and scission concentration also does not change across a 1 mm depth of samples.^48^ According to Khabbaz et al., the oxidation of LDPE samples at 60 °C results in almost the same absorbance of the carbonyl peak on the surface and in the bulk.^46^ Since samples in this study are 1 mm thick, the measurement of the carbonyl index using the FTIR-ATR method can be used to compare samples with minor interference for shorter-aged samples.

The FTIR spectra are presented in Figure 1 before thermal oxidation and after 20 days in an oven at 90, 100, and 110 °C. From the FTIR spectrum of nondegraded LDPE samples, the characteristic peaks for polyethylene can be distinguished at approximately 2915, 2845, 1465, and 720 cm^–1^. These peaks are assigned to the methylene groups (–CH_2_–). In the spectrum for degraded samples at 90, 100, and 110 °C, an additional absorption band in the range of 1850–1600 cm^–1^, indicating the oxidation of polyethylene samples because it is assigned to the carbonyl band, was observed.^49^ For the samples after oxidation for 20 days at 110 °C, the peak at 1170 cm^–1^ is also visible, related to the C–O band stretching vibration in esters.^50^ To further investigate this, data related to the carbonyl band were carefully analyzed, especially the intensity of this peak. This procedure helped calculate the carbonyl index (1), a quantitative and commonly applied parameter to evaluate the thermal oxidation rate. Figure 3 presents values of the carbonyl index correlated with the time of thermal oxidation and variables from temperatures of 90, 100, and 110 °C.

**Figure 1:**
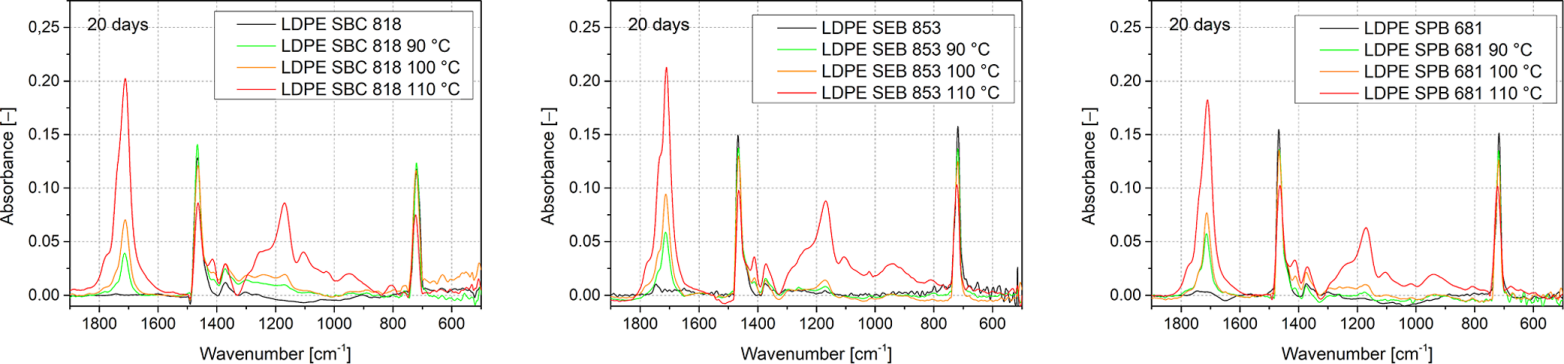
FTIR spectrum of thermally aged bioPE after 20 days at 90, 100, and 110 °C in the region of oxidation products’ presence.

Based on Figure 2, the material undergoing the fastest thermal oxidation is LDPE SEB 853 because just after 2 days at 90 °C, the carbonyl index increases from 0 to 0.27. The evaluation of the whole time interval shows that the growth of CI is not constant, considering the previously mentioned material; the more linear waveform of increase in the carbonyl index is for LDPE SBC 818 degraded at 90 and 100 °C, but the first appearance of the carbonyl peak is just after 5 days. The LDPE SPB 681 sample has a similar growth of carbonyl index to LDPE SBC 818 but results in lower values. Thermal oxidation at 100 °C for all samples led to a similar oxidation route as at 90 °C, with proportionally higher CI, except for SPB 681, where oxidation was observed just after 2 days at 100 °C and after 5 days at 90 °C. The oxidation at 110 °C is more rapid than for other evaluated temperatures and accelerates the growth of the carbonyl index as the aging time increases. It is worth noting that the 110 °C temperature is near the melting point of all three polyethylenes. This type of oxidation is more like in a melt state than solid like for the other two temperatures and is more likely to cause molecular enlargement and cross-linking.^51^ Due to the long-term oxidation processes, the carbonyl index for LDPE SBC 818 and SPB 681 decreases after 20 days of aging at 110 °C compared to 16 days of aging under these conditions. This behavior can be attributed to the partial degradation of materials, which results in volatilization with the formation of VOCs within the CH_3_ group.^52^ This degradation can cause a decrease in the intensity of the CH_3_ peak, the reference for carbonyl index calculation. The chosen reference is supposed to be resistance to thermal oxidation, but the sudden decrease in the intensity of the methylene group peak is observed for SBC 818 and SPB 681 after 16 days under 110 °C, and this results in a higher carbonyl index, not corresponding to the observed continuous increase in the intensity of the carbonyl peak.

**Figure 2:**
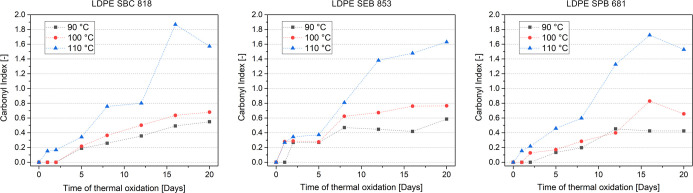
Carbonyl index as a function of the time of thermal oxidation.

Except for the calculation of the carbonyl index, this characteristic peak can be deconvoluted to thoroughly analyze what kind of oxidation products occur after thermal oxidation. The peak, whose maximum is located between 1800 and 1650 cm^–1^, is usually the result of overlapped absorption bands.^53^ The characteristic chemical products that can be distinguished are carboxylic acids, ketones, esters, peresters, and lactones.^54^ Figure 3 presents the results of peak deconvolution analysis for samples thermally oxidized for 20 days at 110 °C. Other examples of deconvolution are presented in the Supporting Information (Figure S2) for samples thermally oxidized for 20 days at 90 and 100 °C. The quantity of the obtained deconvoluted peaks is different for each bioLDPE grade. The oxidation products detected by FTIR spectroscopy differ between samples and depend on the oxidation time and temperature. Each deconvoluted peak is matched to the corresponding chemical bond.^27,54–56^

**Figure 3:**
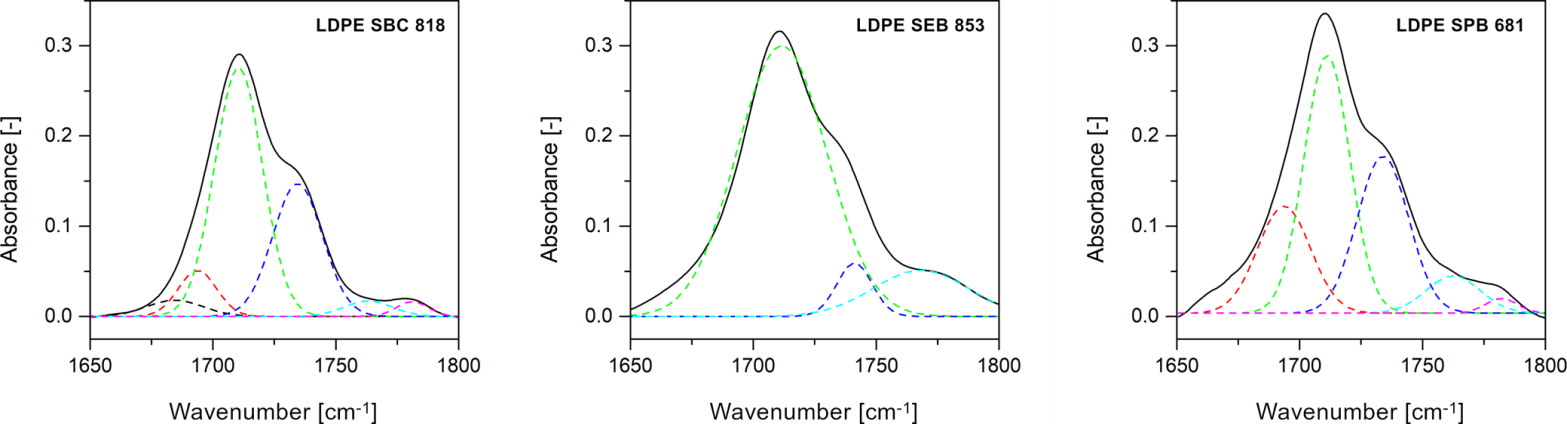
Deconvoluted carbonyl bands after thermal oxidation for 20 days at 110 °C.

All of the prominent peaks with physical equivalents are listed in Table 2. The Lambert–Beer law was used to estimate carbonyl compound concentration to better segregate all of the oxidation products based on time and temperature. The four main groups of carbonyl compounds were separated: ketones, esters, acids, and lactones. The noticeable peresters were classified into the ester group since they are equivalent to carboxyl esters.^57^

**Table 2:** Wavenumber and Oxidation Product of Nine Distinguished Peaks from the Carbonyl Band

No.	Wavenumber [cm^–1^]	Oxidation products
1	1686	ketones with aldehydes α, β unsaturated
2	1697	γ-ketoacids, keto group
3	1713	carboxylic acids
4	1720	ketones
5	1735–1750	esters
6	1757	carboxylic acid anhydride
7	1761	carboxylic acid (isolated) anhydride
8	1780	peresters
9	1783	γ-Lactones

Molar concentrations of carbonyl species are presented in Figure 4, diversified based on biopolyethylene grade and thermooxidation temperature. All distinguished carbonyl compounds after the deconvolution of the FTIR peak were split into 4 groups of compounds: ketones, esters, acids, and lactones. In this particular instance, the Lambert–Beer law has been proposed to evaluate molar concentrations from spectroscopic measurement. This equation represents a linear relationship between absorbance, the concentration of the absorbing substance, and the distance the light travels through the sample.^58^ The ATR method tends to deviate from the Beer–Lambert law, particularly when dealing with strong absorptions. Although it performs effectively for weak absorptions (where the absorption index remains below 0.1), it encounters difficulties with stronger absorptions commonly found in organic and biological materials, such as those involving C–O, C=O, or O–H functional groups.^59^ However, the measurement of polyethylene in transmission mode is difficult because only extremely thin layers can be analyzed using transmission methods, 20–50 μm.^60^ The previously mentioned penetration depth in the ATR method is also one of the limitations of using the Beer–Lambert law; it requires careful handling and corrections to ensure meaningful data, particularly for quantitative analysis. To avoid the impact of these restrictions, the integrated absorbance can be used instead of peak absorbance; it is very effective for asymmetric bands and in cases when the ratioing of overlapping peaks occurs.^59,61^

**Figure 4:**
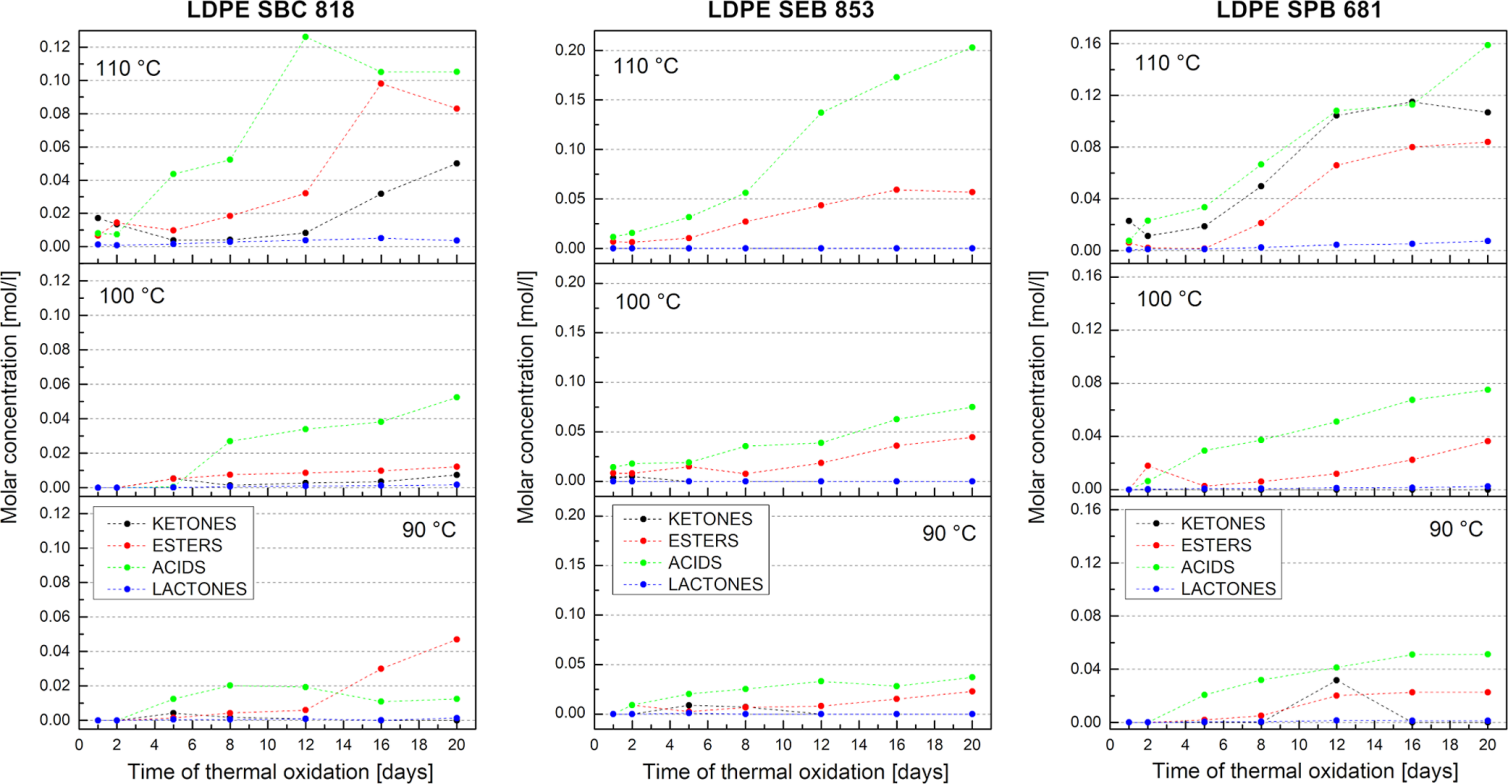
Molar concentration of the four main carbonyl compounds after oxidation in three bioLDPE grades as a function of time and temperature.

The first noticeable thing is the minor concentration of lactones, exactly γ-lactone, only for samples LDPE SBC 818 and LDPE SPB 681 at 90, 100, and 110 °C; the LDPE SEB 853 sample did not exhibit formation of γ-lactones during thermal oxidation. The lactone amount is approximately 10 times smaller than the rest of the oxidation products. The changes in molar concentrations of carbonyl species are nonmonotonic with time and temperature. This phenomenon is caused by both an increase or decrease in the carbonyl compound peak absorbance area and the disappearance or appearance of distinguished peaks. Figure S3 presents charts with marked information regarding the time and temperature at which thermooxidation peaks occur. As can be seen, nine different carbonyl compounds were encountered after the deconvolution of the carbonyl peaks (Table 2). Other carbonyl compounds are visible depending on the polyethylene grade. The most significant difference is related to the appearance of a ketone at 1720 cm^–1^. This compound appears only for LDPE SBC 818 oxidized at 100 °C for 5 days and at 110 °C for 1 and 2 days; another sample with captured ketones at 1720 cm^–1^ is LDPE SPB 681 after oxidation for 1 day at 110 °C. In the remaining samples, ketone compounds were observed at 1690 cm^–1^, identified as ketones with aldehydes, α, β unsaturated. Additionally, a ketone group was detected at 1697 cm^–1^, corresponding to γ-ketoacids in SBC 818, which had undergone oxidation at 110 °C for 20 days. During the oxidation of LDPE SBC 818 at 110 °C, the breakage of the carbonyl group bond in ketones (XI–XII) to form carboxylic acid (IX–X) occurs after 5 days; this results in the reduction of ketones at 1720 cm^–1^ in favor of carboxylic acids. This specific behavior shifted the carbonyl peak’s highest absorption to the carboxylic acid’s deconvoluted peak, previously described by Gardette et al.^29^ for the photooxidation process. Ketone oxidation products for LLDPE thermally oxidized represent 130% of the carboxylic acid concentration. On the other hand, during photo- and gamma-radiation-initiated oxidation, the ketones constitute 26% and 80% of the carboxylic acid concentration, respectively.^62^ For samples oxidized at 90 and 100 °C, the formation of ketones is negligible. LDPE SBC 818 aged at 110 °C exhibits an increasing concentration of ketones lower than carboxylic acids concentration, similar to LDPE SPB 681, but in this case, the concentration is approximated; however, for SEB 853 at 110 °C, no ketone compounds occur. The replacement of ketone oxidation products for carboxylic acids resembles the characteristics of photooxidation of polyethylene, where both compounds occur but with slightly higher concentrations of acids.^29^ The same conditions were noticed for LDPE samples aged in natural weathering conditions.^28^ Another compound in small concentrations in addition to lactones is perester, which is observed only for LDPE SEB 853. Detecting components like peresters is barely possible above 1740 cm^–1^. The lack of the presence of this compound in LDPE SBC 818 and LDPE SPB 681 cannot clearly state that this carbonyl species was not formed during oxidation. Other components constituting products of oxidation of LDPE SEB 853 that are present throughout the whole oxidation time at 100 and 110 °C with increasing concentrations are acids and esters. It is reported that ketones and carboxylic acids are formed at the first stages of oxidation, and with increasing oxidation time, the ester groups are more likely to be found in further steps.^63,64^ For the LDPE SPB 681 sample, the esters, ketones, and carboxylic acids are observed simultaneously with a more rapid increase in concentrations of esters and acids. Formation of esters (XV) during thermal oxidation is found to be present in a solid-state case.^63^ The highest concentrations of esters are in LDPE SBC 818 oxidized at 110 °C, and a tendency to increase their concentration over time is observed for all samples. For SBC 818, above 16 days of thermal degradation at 90 °C, the ester concentration surpasses the acid concentration. The ester groups are found to appear in later stages of oxidation due to the reactions between carboxylic acids and alcohols to form ester groups.^63^ The difference between samples, except for the elongation of the oxidation time, is the temperature. The concentration of oxidation products at 90 and 100 °C remains at a similar level; increasing the oxidation temperature to 110 °C causes a several-fold increase of carbonyl compound concentration up to 0.20 mol/L of carboxylic acids for SEB 853. After 1 day, samples oxidized at higher temperatures contain carbonyl compounds. It is in agreement for all samples at 110 °C and additionally for LDPE SEB 853 at 100 °C. The polymer’s oxidation depends on oxygen diffusion, which is limited at lower temperatures.^65^ The sensitivity to thermal oxidation is also related to the molecular weight distribution of polyethylene, and materials with higher dispersity exhibit a decline in resistance to thermal oxidation.^66^ However, LDPE SBC 818 with the highest dispersity does not have the highest values of oxidation parameters when analyzing the carbonyl index and molar concentrations of carbonyl compounds.

### 4.3 X-ray Analysis

Analysis of the X-ray diffractograms (Figure 5) for samples before and after aging shows two distinct crystalline peaks between 2θ = 21°–22° and 2θ = 23.5°–24°.^67^ The peaks are correlated with the crystallographic planes of orthorhombic crystals, which are (110) and (200), respectively.^68^ They are both characteristic of polyethylene.^69^ The diffractograms present the decrease in the intensities of the peaks with an increase in the oxidation time; the intensities of these peaks decrease most significantly for samples aged at 110 °C.

**Figure 5:**
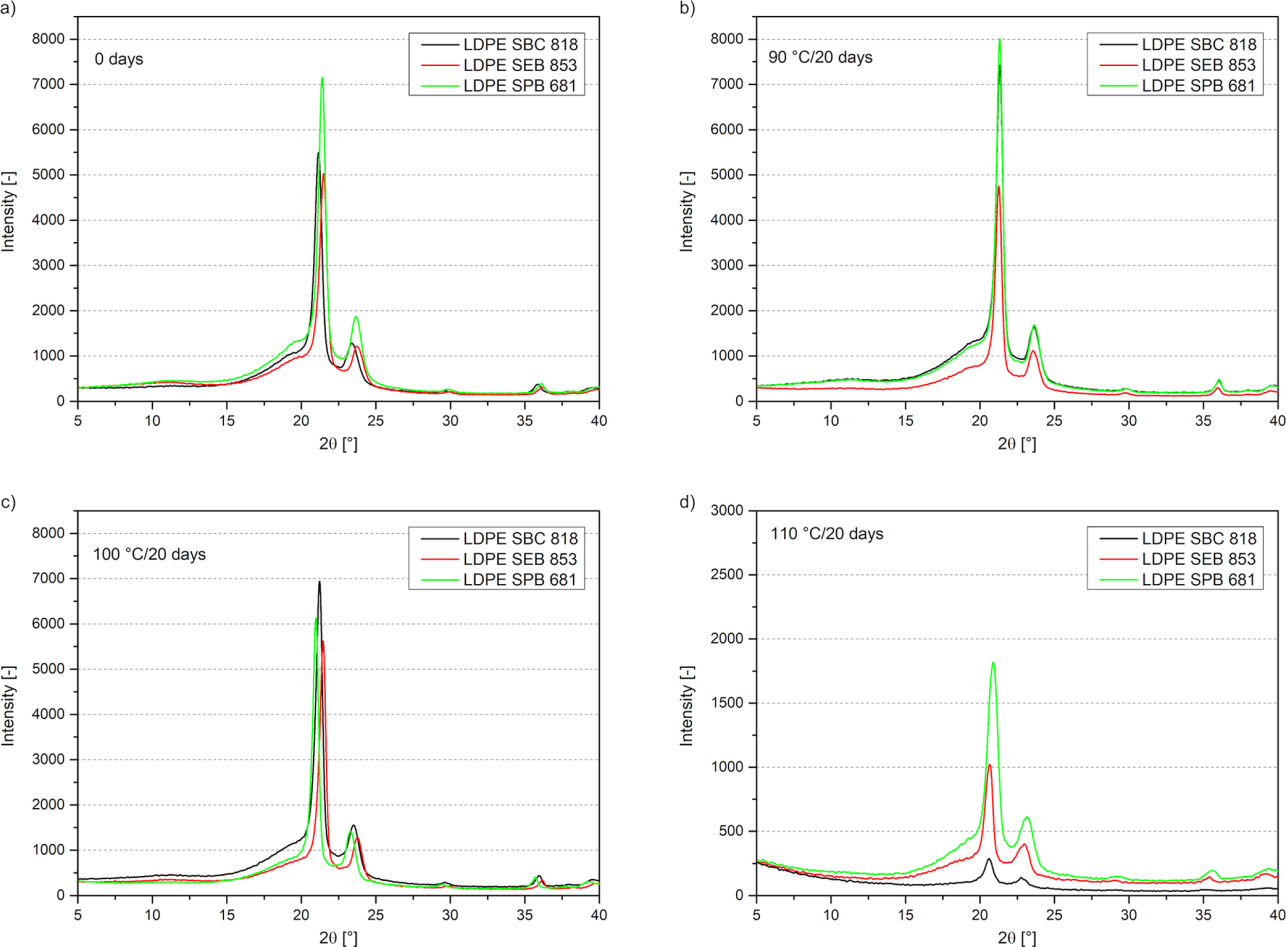
Diffractograms of aged polyethylene samples before thermal oxidation (a) and after 20 days at 90 °C (b), 100 °C (c), and 110 °C (d).

This decrease in intensity is correlated with the reduction of crystallinity (Figure 6). The oxidizing atmosphere causes the transition from crystalline to an amorphous phase. This transition was observed by Han et al.^70^ for thermally aged cross-linked HDPE in the air-circulating oven at 150 °C for 1000 h. They recognized this behavior in the presence of oxygen. In the same way, aged cross-linked HDPE in vacuum did not exhibit this phenomenon. The HDPE pipes degraded in the sunlight for 24 h also have lower intensities of crystalline peaks and degree of crystallinity than before degradation, but the decrease is not this significant.^71^

**Figure 6:**
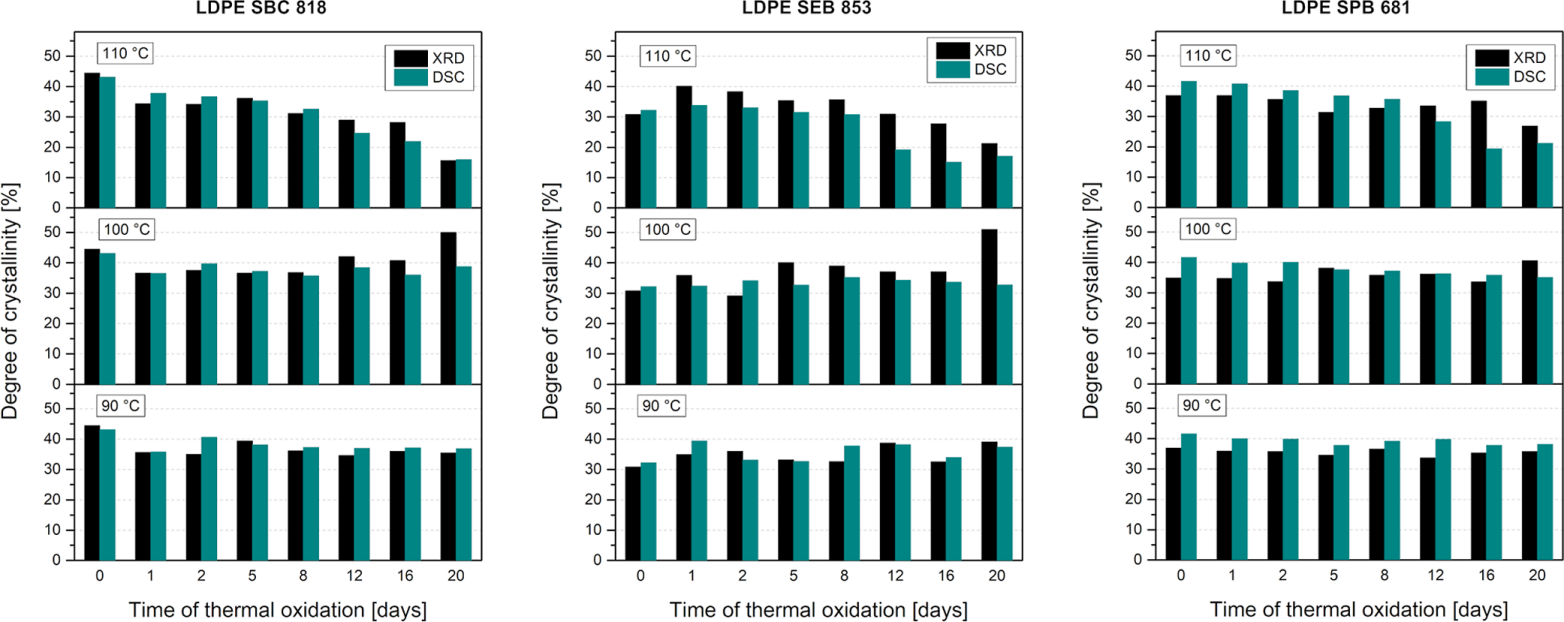
Crystallinity of thermally oxidized LDPE samples measured using DSC and XRD methods.

Considering the change in the crystallite size of polyethylene after thermal oxidation, the most significant differences are between materials degraded at 110 °C. Oxidation at 110 °C causes a reduction in the crystallite size. The most significant decrease is observed for LDPE SBC 818; after 12 days of aging, the average crystallite size was almost 25% lower than before the oxidation. The crystallite size presented in Figure 7 is the average value of crystallite size calculated for two distinguished crystalline reflections. This size from the Scherrer equation for all samples in any aging conditions exhibits a higher value for reflection (110); the same characteristic is observed in the literature.^45,72^ The previously mentioned decrease in the degree of crystallinity is connected with the reduction of crystallite size. On the other hand, the UV-aged LLDPE samples exhibit an increase in the crystallite size.^44,45,73^ It is typical for UV-degraded polyethylene and is caused by the reorganization and recrystallization of small chains formed after the chain scission reaction. The analysis of the FTIR spectra suggests that the degradation behavior is similar to the one caused by UV radiation, and even though the experiment conducted by Hsu et al.^45^ also showed comparable oxidation levels, the crystallinity of UV-aged petrochemical LDPE differs from that of biobased LDPE. The decrease in the crystallite size can occur for cross-linked polyethylene, moving from molten to solid state, where the reorganization and chain folding occur during cooling.^74,75^ A decrease in crystallite size is observed only for samples thermally aged at 110 °C; this temperature is very close to the melting point of all three bioPEs. Highly elongated time of staying at an elevated temperature for samples can cause phenomena occurring during the cooling of polymers from the molten state.

**Figure 7:**
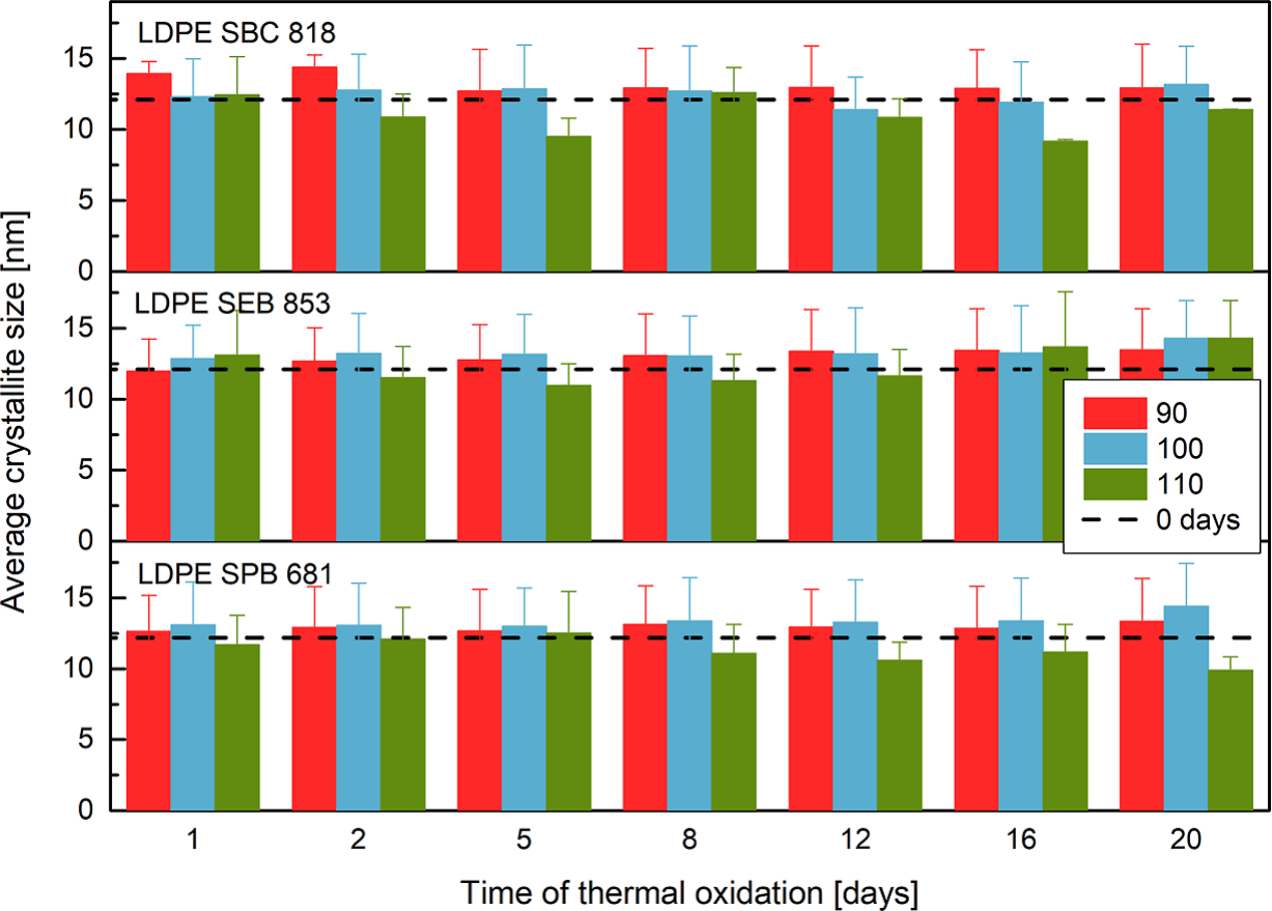
Average crystallite size.

### 4.4 Differential Scanning Calorimetry

The previously mentioned degree of crystallinity measured by X-ray diffraction was also analyzed using differential scanning calorimetry. In Figure 6, these parameters are present, and two methods are compared. There are slight differences in the degree of crystallinity; the value calculated by the DSC method is higher for almost all samples, but the characteristics of the changes are the same for both methods. The degree of crystallinity decreases for samples aged at 110 °C with the growth in oxidation time. It is usually observed from DSC that the thermo- or photodegraded polyethylene exhibits an increase in the degree of crystallinity due to the recrystallization caused by chain scission.^19,44,71,73,76,77^ The amorphous phase is more susceptible to aging, and the chain scission reaction results in low-molecular-weight products that tend to recrystallize. The second melting DSC endotherms are collectively presented in Figure 8. The only mentioned possibility of the decrease of the degree of crystallinity after thermal degradation is observed in cross-linked HDPE; after 1000 h at 150 °C in air, the melting peak disappears, and the crystallinity calculated from XRD decreases.^70^ The disappearance of the endotherm peak is observed for LDPE SBC 818 at 110 °C. This effect is also correlated with the decrease of the melting temperature by almost 30 °C for LDPE SBC 818 after 20 days of thermal degradation at 110 °C, resulting from cross-linking of the polymer.^78^ For LDPE SEB 853 and LDPE SPB 681, the broadening of the endotherm peak and the appearance of a new peak at lower temperatures, called shoulders, were noted. The thermal degradation at 90 and 100 °C did not cause those shifts in a DSC thermogram. The melting temperature for unaged samples is 108.1, 110.9, and 111.1 °C, respectively, for LDPE SBC 818, LDPE SEB 853, and LDPE SPB 681, which means that the degradation at 110 °C is approximately at the melting point. Below that temperature, no evidence of thermal degradation is visible from the DSC thermograms. The previously mentioned work performed by Gong et al.^71^ about sunlight-degraded HDPE pipes shows a decrease in the degree of crystallinity measured by X-ray diffraction and DSC, and they also exhibit cross-linking mechanisms in the UV degradation of those pipes. The main conclusion drawn from this analysis concerns the degradation mechanism, in which the cross-linking exceeds the chain scission reaction for degradation at 110 °C.

**Figure 8:**
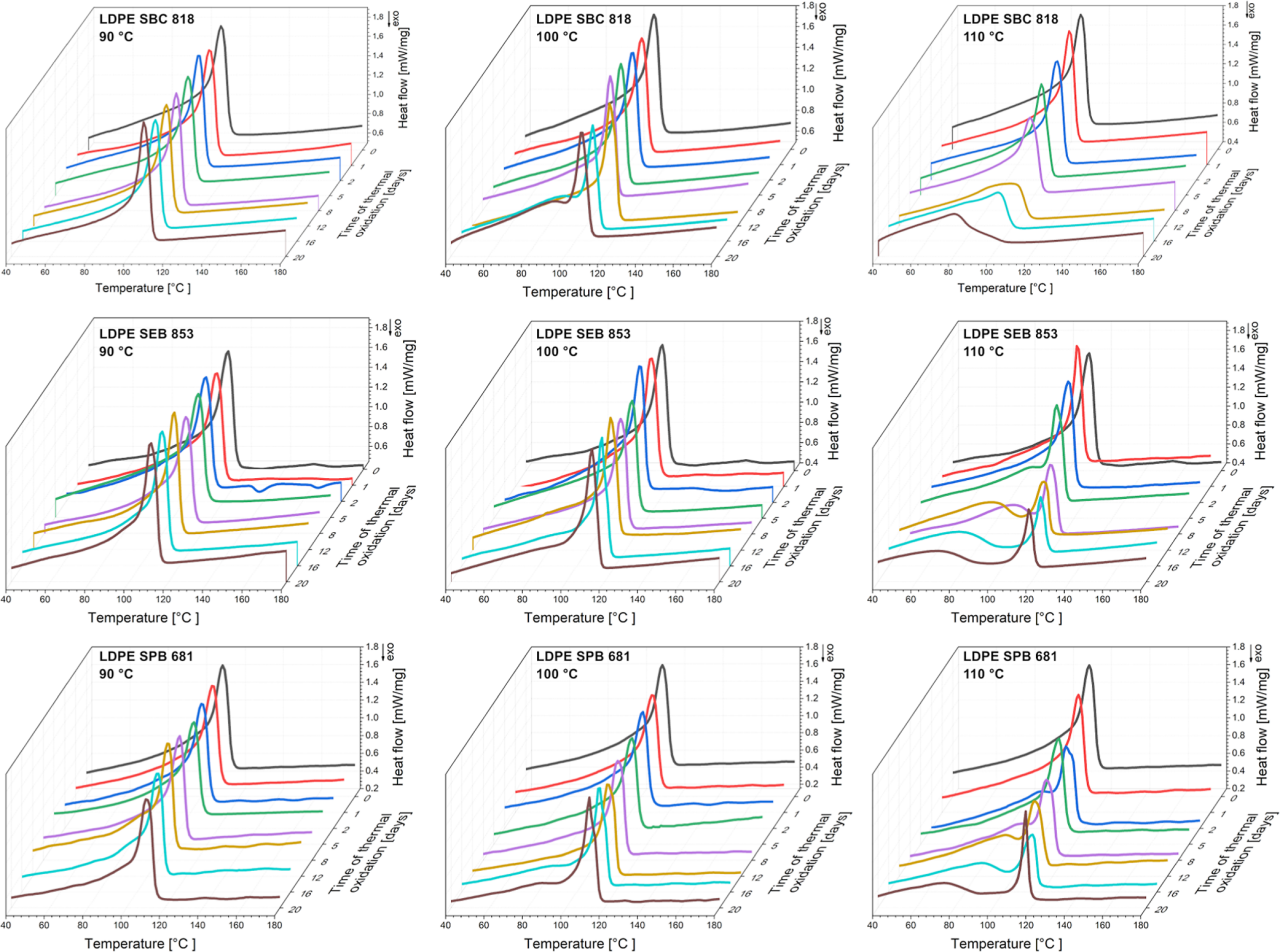
DSC second melting thermograms for three bioLDPE grades after thermal degradation.

### 4.5 Oxidation Induction Time and Temperature

The analysis of oxidation induction time is a method to investigate the resistance to oxidation of thermoplastic polymers in the molten state and is a useful tool for determining the efficiency of introduced stabilizers.^32,79^ OIT is measured to define the time after material oxidation occurs, which is measured as an exothermic effect after changing the atmosphere from inert (nitrogen or argon) to oxygen. The results of the comparative analysis of the oxidation stability of the tested series of low-density polyethylene before the aging process are summarized in Table 3. All analyzed materials are characterized by low resistance to oxidation, which poses a problem from a measurement implementation point of view. Taking into account the guidelines presented by Schmid and Affolter^32^ for the analysis of polymers with low oxidation resistance, measurements were carried out at three temperature values: 170, 180, and 190 °C. Based on the tests, it can be concluded that all LDPE series before aging are characterized by comparable resistance to oxidation in the molten state. The most significant differences between LDPE grades were observed during measurements using the lowest temperature of 170 °C; in this case, the longest OIT was recorded for SEB 853. The obtained results are consistent with the values presented previously in the literature. Babaghayou et al.^76^ analyzed unstabilized LDPE with a result in 18 min of OIT at 180 °C. Other investigations showed that the nonstabilized LDPE has an OIT of 2 min, and biobased HDPE has 2 min.^80,81^ The material exhibiting the lowest oxidation induction time at 170 and 180 °C is also characterized by the highest dispersity. The broad molecular weight distribution of polyethylene is associated with increased sensitivity to oxidation in thermally induced conditions.^82^

**Table 3:** Oxidation Induction Time (OIT) of Three Biobased Polyethylene Grades before Aging

Material	OIT_170 °C_ [min]	OIT_180 °C_ [min]	OIT_190 °C_ [min]
LDPE SBC 818	21.1 ± 1.0	10.5 ± 0.9	5.2 ± 0.1
LDPE SEB 853	24.8 ± 0.6	10.6 ± 0.2	5.1 ± 0.1
LDPE SPB 681	22.9 ± 0.5	11.8 ± 0.1	5.2 ± 0.1

The oxidation onset temperature (OOT) determines the temperature at which the onset of the DSC curve is noticed during heating in an oxygen atmosphere. These temperatures are marked as the onset of thermal degradation in the oxidative atmosphere.^83^ No matter what temperature, the thermal aging of biobased polyethylenes causes a decrease in the level of the OOT compared to non-aged samples. The values of OOT are presented in Figure 9 as a function of thermal oxidation time for each material and three different temperatures. The highest decrease is observed for samples aged at 110 °C, with a sudden increase after the samples reached the lowest bottom values. The different thermal oxidation times were recorded for each material: 5 days for LDPE SBC 818, 8 days for LDPE SEB 853, and 5 days for LDPE SPB 681. The aging of polyethylenes at lower temperatures (90 and 100 °C) results in a constant decrease of the OOT with an increase in thermal oxidation time. The lower oxidation onset temperatures are observed for samples aged at 100 °C than for 90 °C, but the lowest are after oxidation at 110 °C before the sudden rise. The onset oxidation temperature decreases with an increase in remelting cycles by approximately 15 °C for LDPE samples stabilized with artificial antioxidants.^84^ The OOT for biobased polyethylene lowers its value with the elongation of thermal oxidation time, which is also about 15 °C, but only for samples aged at 90 and 100 °C. A temperature set of 110 °C leads to a decreased OOT of 25.4 °C for LDPE SBC 818, 20.7 °C for LDPE SEB 853, and 18.2 °C for LDPE SPB 681 .

**Figure 9:**
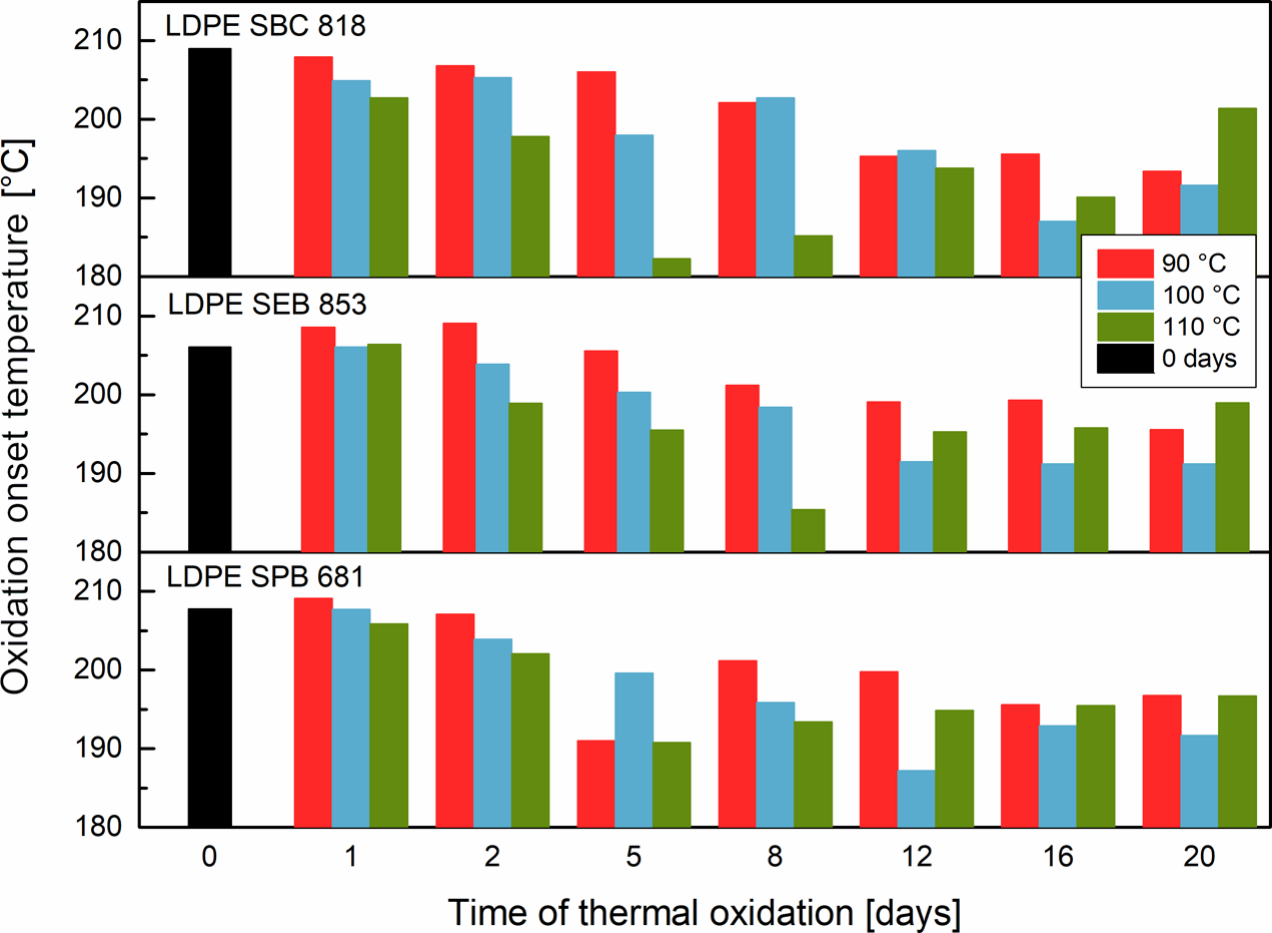
Changes in the onset oxidation temperature after thermal oxidation.

**Figure 10:**
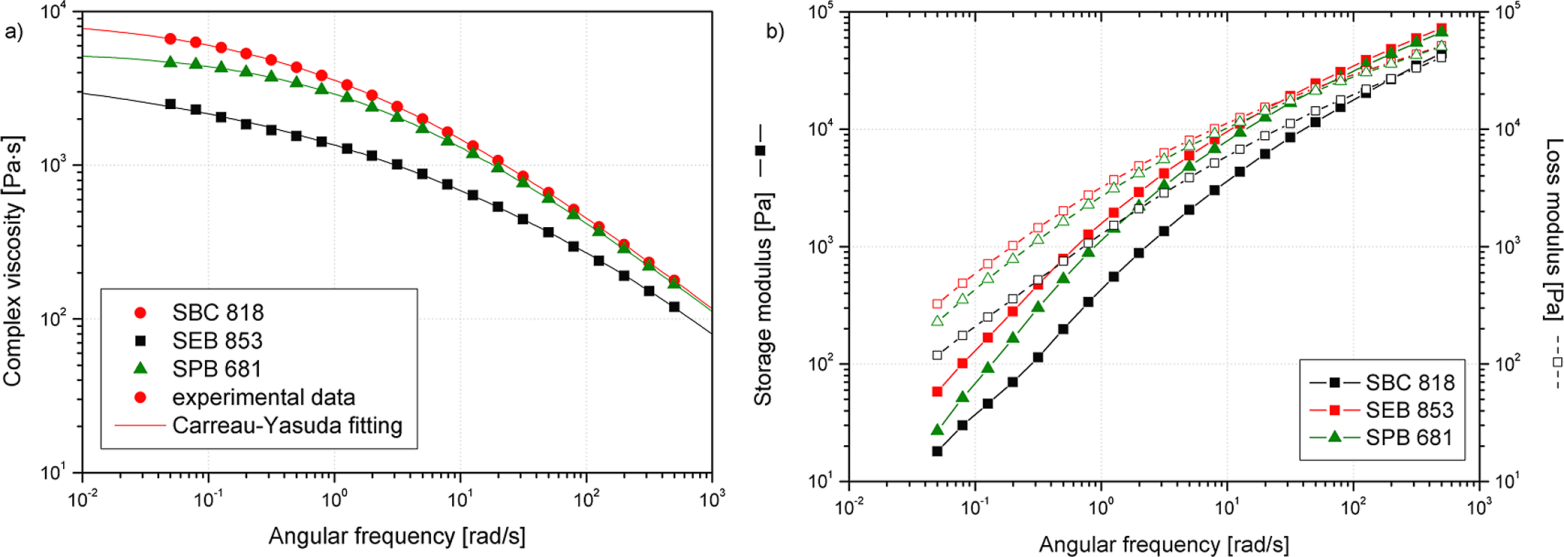
Complex viscosity (a) and storage and loss modulus (b) vs angular frequency curves of the studied polyethylene grades.

### 4.6 Rheological Analysis of Biobased Polyethylene Structural Changes

Polyethylene’s rheological behavior is correlated with its molecular weight and macromolecular structure, including long chain branches (LCB) content.^85^ Rheological measurements were used in this study to indirectly describe the macromolecular structure of the analyzed varieties of biobased low-density polyethylene grades. In addition, this analysis was used to describe in detail the changes in this structure due to thermal aging.

The crossover point of *G’* (storage modulus) and *G″* (loss modulus) vs ω (angular frequency) curves is related to the dispersity and molecular weight of polyethylene.^86,87^ As Ansari et al.^87^ described, the lower values of *G*′=*G″* at the intersection point of the curves are characteristic of polyethylene grades with lower dispersity. At the same time, a location of the crossover point toward lower angular frequency values is observed for higher-molecular-weight polyethylene varieties;^88^ however, it may also be the result of the presence of an increased number of long side branches, causing an increase in elasticity of the melt.^86^ In the literature, the correlation of rheological parameters determined by fitting rheological models to measurement data with changes in molecular weight was described.^89^ SBC 818 and SPB 681 revealed the complex viscosity curves characterized by Type-I viscosity function, while SEB 853 showed a minimum in the slope, and a so-called S-shaped viscosity function^90^ was noted (Type-II) (Figure 10). Therefore, regarding the possible occurrence of changes in the course of the curve in the terminal region, it was not decided to directly correlate the results obtained by HT-SEC with the rheologically determined weight-average molar weight due to the possibility of deviation of the results for branched polymers without creep experiment supplementation.^90,91^ At the same time, fitting the Carreau–Yasuda model may be useful in the qualitative assessment of the occurrence of LCB. The rheological data presented in Table 4 show that the used polyethylene grades differ significantly. Distinct variations in the shortened relaxation time and noticeably lower η_0_ of SBC 818 indicate a lower amount of LCB of this LDPE variety than SEB 853 and SPB 681.^92,93^ As Tabatabaei et al.^94^ described, the longer relaxation time for polymers with higher LCB is connected with changes in the stress relaxation mechanism and more complex reptation mechanisms induced by entangled long-chain branches. Moreover, in the case of SEB 853, an additional curvature of the complex viscosity curve below 0.01 rad/s can be observed, which is related to the presence of LCB, which results in the appearance of additional relaxation modes and changes the relaxation spectrum.^90^

**Table 4:** Zero Shear Viscosity η_0_ and Data of *G*′ = *G*″ Crossover Point Relaxation Time θ

Material	η_0_ ^a^ [Pa·s]	λ^a^ [ms]	*a*^a^ [-]	*R*^2^^a^ [-]	ω and *G*′ at *G*′ = *G*″^b^ [rad/s; Pa]	θ^b^ [ms]
LDPE SBC 818	4961	5.40	0,2158	0.998	214.0; 27,830	4.67
LDPE SEB 853	9166	762.89	0,4352	0.999	26,74; 17,360	37.40
LDPE SPB 681	5533	538.68	0.5186	0.999	42.85; 19,860	23.34

a Data obtained from the Carreu–Yasuda model fitting.

b Data obtained from *G*′ = *G*″ crossover.

Figure 11 presents complex viscosity curves for polyethylenes aged at 90, 100, and 110 °C for up to 20 days. Two trends in the course of the viscosity curves can be noted. The first is associated with a decrease in molecular weight due to degradation, resulting in a lower viscosity over the entire angular frequency range. This effect is visible for samples thermally aged at 90 °C. In the case of the series exposed to 100 °C under oxidizing conditions, the decrease in viscosity was more rapid. The disappearance of the complex viscosity curve transition to the first Newtonian range was also observed for samples subjected to long aging times, which is related to the formation of spatially cross-linked LDPE structures. This effect is evident for 110 °C aged materials; however, it could also be noticed for LDPE aged at lower temperatures, for series aged over 16 days (90 °C) and 8 days (100 °C). Interestingly, based on the |η*|(ω) curves, it can be supposed that for the samples subjected to the thermooxidation process with a temperature of 100 °C, the effect of intense chain scission occurred, with the simultaneous beginning of the cross-linked structure formation process. The process of polyethylene cross-linking due to the long-term temperature influence may result from two different mechanisms. The first reaction is related to the secondary alkyl reaction, leading to junctions created by covalent bonds joining tertiary carbon atoms. The second mechanism highlighted in the literature is associated with coupling the vinylidene group with a skeleton alkyl, resulting in the formation of bonds between two tertiary carbons separated by a CH_2_ group.^95^ Nonmonotonic changes in viscosity observed for material samples aged at 100 °C for 16 and 20 days are probably due to very intense degradation, with the simultaneous beginning of the formation of spatially cross-linked structures.

**Figure 11:**
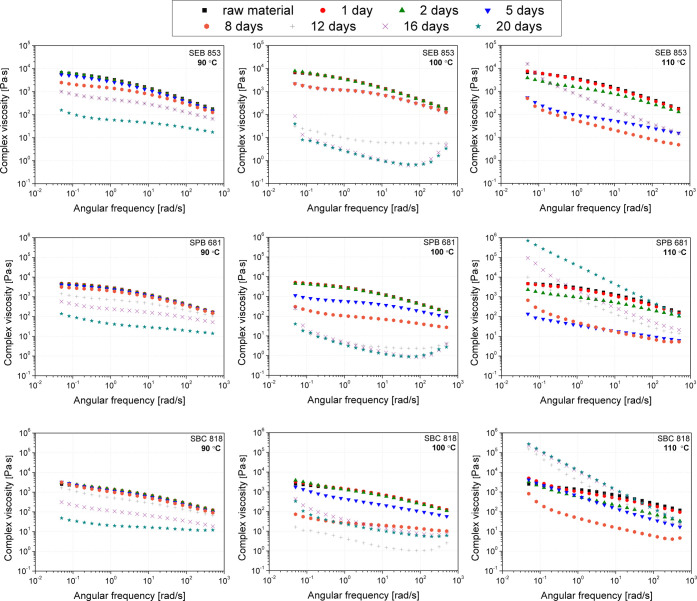
Complex viscosity curves of three grades of biobased polyethylene subjected to thermal aging.

Cole–Cole plots illustrate changes in the imaginary viscosity part (η″) in terms of the real part of the viscosity (η′), assessed by the oscillatory rheological test. This type of rheological data interpretation allows the assessment of the viscoelastic properties of polymers as well as interactions and complex mechanisms in polymer blends or composites.^96,97^ The right part of the graph shows information about viscous behavior, which often translates into accompanying longer relaxation times for more viscous deformations.^98,99^ However, measurements made at a high angular frequency and, in the case of non-cross-linked polymers, allow correlating η′ values with the transition from viscous to elastic behavior. Observed in Figure 12, especially for the SEB 853 and SPB 681 series subjected to thermal aging at 90 and 100 °C, the reduction of parabolas with the simultaneous lowering of η″ is due to significant degradative changes, including chain scission from polyethylene without changing their character of the single semicircle.^100,101^ This is related to the faster relaxation mechanism of polymer chains of degraded polyethylene, during which the movements of macromolecules with a reduced length became easier.^99^ The smooth semicircle shape of Cole–Cole plot disorders is usually associated with limited mobility of macromolecules, including cross-linking,^101,102^ incompatibility of polymer mixtures,^100,103^ or the formation of a physical network of interactions of the filler dispersed in the matrix.^104,105^ In the considered case, the change in the shape of Cole–Cole curves was related to cross-linking behavior induced by thermal treatment and oxidation of polyethylenes. This effect is most pronounced for the polyethylene grade bimodal characteristic of molecular weight distribution and the highest dispersity (SBC 818) and least noticeable for SEB 853, which, as suggested by rheological data, is characterized by the most branched structure. It was noticed that for the SBC 818 sample, the effect of the curve inflection in the range of higher η′ values is observed after the shortest aging time and the lowest temperature (1d; 90 °C), while for the case of SEB 853, this effect occurs only after partial degradation of the polymer. Macromolecules subjected to defragmentation form partly cross-linked structures. However, this is not the dominant rheological effect in the aging temperature range of up to 100 °C for SEB 853 and SPB 681. Only in the case of aging at 110 °C is the formation of a spatially cross-linked structure observed for all samples’ aging times exceeding 2 days. In conclusion, based on the rheological analysis, it can be stated that the decreasing degree of branching, dispersity, and increasing molecular weight of biobased polyethylene increase its tendency to form cross-linked structures. In addition, the aging temperature played a dominant role in changing the nature of the phenomena from degradation and chain scission to a progressive cross-linking process. The cross-linking of LDPE causes a decrease in elongation at break and an increase in maximum tensile stress due to decreased crystallinity and a smaller length of segments available for stretching; however, the increase in tensile stress is caused by stronger chemical cross-linking bonds than intermolecular forces.^106^ Liu et al.^106^ provided a study about peroxide cross-linking, and even the lowest amount of chemical cross-linking agent in LDPE caused no significant flow characteristics of the polymer, transforming it into a more rubber-like material. The cross-linking leads to chemical and physical cross-links forming between polymeric chains, in which chemical cross-links are stronger, and the constraint on the polymer’s elongational behavior becomes more pronounced.^107^ Cross-linked polyethylene substitutes PE with improved insulating capacity and thermal resistance up to 120 °C for cable insulations, pipes, and plumbing for hot water distribution.^108^

**Figure 12:**
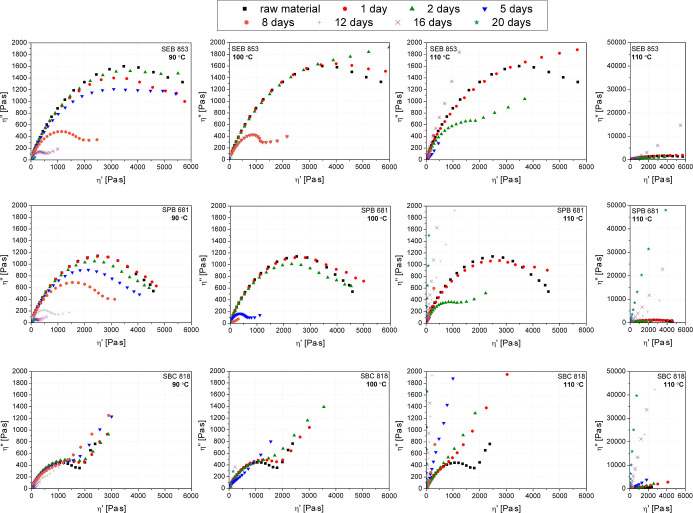
Cole–Cole plots of three grades of biobased polyethylene subjected to thermal aging.

### 4.7 Color (Yellowness Index)

Additional analysis of the sample color was performed. After thermal oxidation, samples have exhibited color changes, mainly in the *b* parameter of the CIELab color parameters. The b values differ from (−) for blue to (+) for yellow. Figure S4 presents the Supporting Information on changes in the value of *b* for different thermal oxidation durations. The extended time of thermal aging at 110 °C causes an increase in the value of *b*, which means the yellowing of samples. Another method to investigate changes in the color of samples is to calculate the yellowness index (*YI*). It is a well-known variable used to measure the effects of degradation of polyethylene.^109,110^ The higher the value of this index, the more degraded the samples usually are. The changes in the parameter *b* become visible only after thermal degradation at 110 °C, and the same applies to the YI. The courses of those two curves are close to each other. The highest values of *b* and *YI* are for LDPE SBC 818, and the samples’ yellowing starts earlier than for LDPE SEB 853 and LDPE SPB 681. The *YI* for LDPE SBC 818 is more than two times higher than the values for the two other materials. This can be linked with the carbonyl index, which has the highest value for the LDPE SBC 818 after 16 days of oxidation at 110 °C. In Figure S4, the values of *b* and *YI* are presented, as well as the graphical representation of colors as a background of the graph.

### 4.8 Oxidation Analysis of Samples by Energy-Dispersive Spectroscopy

The oxidation of polyethylene induced by UV radiation and elevated temperature exposure is associated with a variation in the intensity of the changing profile of the chemical structure of the polymer within the material, intensifying from the outer layer.^44,111–113^ In this research, an additional attempt was made to determine the nature of the oxidation phenomenon (surface or volumetric). For this purpose, preparations from aged polymer samples were made by using a microtome, and the cross sections of the samples were analyzed using the SEM-EDS technique. Our previous studies describing the structural changes of polyurea-based composite coatings exposed to UV radiation proved the possibility of qualitative assessment by this method and the depth of the oxidized layer.^114^ Figures S5 and S6 (Supporting Information) show EDS mapping of oxygen in a cross-section of aged polyethylene samples after 5 and 20 days of exposure to various temperatures in the air atmosphere. Scanning electron microscopy with energy-dispersive X-ray spectrometry identifies elements of the periodic table;^115^ according to this, black dots presented on SEM images correspond to the presence of oxygen in the cross-section of the sample. The selection of samples for analysis after 5 days resulted from the intensive changes observed using other techniques and differentiating polyethylene grades in their stability. The presented mapping allows us to conclude that the phenomenon of intensification of the oxidation process, observed qualitatively by increasing the intensity of the observed oxygen-rich areas, increases with the higher temperature of aging. The solubility and permeability of oxygen in polyethylene are strictly correlated with the crystallinity of the material since the oxygen solubility can be measured using the equation

9 $$S=\alpha \times S^{*}$$

where *S* is the solubility, S* is the solubility in the amorphous phase, and α is the content of the amorphous part.^116^ The solubility of oxygen depends on the amorphous phase content because oxygen is insoluble in the crystalline part of the polymer. Aging of polyolefins in natural conditions (ambient temperature) yields a homogeneous oxidation level throughout the 1 mm thick samples; however, accelerated aging at 90 °C has led to a higher oxidation level at the surface than the core of the sample.^117^ In both cases, there was no structural gradient in the intensity of the occurrence of oxygen from the outer surfaces for samples after 5 and 20 days of aging. Therefore, it can be concluded that oxygen diffusion accompanied the long-term oxidation process at temperatures exceeding α-relaxation of the LDPE^118^ into the deeper layers and caused uniform oxidation throughout the samples. The difference with the reported results for XLPE, where a gradient of the oxidized layers formed by elevated temperature in the oxidative atmosphere was observed,^111^ is due to the much greater oxygen diffusivity of LDPE.^119^ ATR-FTIR tests performed in this study can be considered representative measurements for the entire sample, which aligns with the conclusions of Wilson,^120^ who described the volumetric oxidation of a polymer subjected to thermal exposure. The observed surface artifacts are related to sample chipping and measurement errors. The most intense changes were recorded for the LDPE SEB 853 samples at lower aging temperatures. However, the most significant impact of temperature on the oxidation process was noted for SBC 818, a polymer with low molecular weight dispersity, in which the highest temperature of 110 °C caused a significant increase in the intensity of oxygen presence in the entire sample volume.

Figure 13 presents the images of cryo-fractured polyethylene samples before and after thermal aging for 20 days at 110 °C. Additional images of samples aged for 5 days, and all three aging temperatures included, are presented in the Supporting Information (Figures S7–S9). Samples before oxidation exhibit a highly folded and developed structure related to a ductile fracture of polyolefins - high roughness and significant plastic deformation confirm this.^121^ After thermal oxidation for 20 days, samples had a much smoother fracture surface with small, thin cracks. In this case, observed structures suggest the presence of brittle fracture along with simultaneous occurrence of crazing.^121^ Thermal oxidation affects the mechanical properties of polyethylene, and with increasing thermal exposure, the material tends to become more brittle.^122^ Analyses of the other SEM images present in Figures S6–S8 conclude that after 20 days of thermal oxidation, SEB 853 and SPB 681 samples exhibit brittle fracture and after 5 days at 110 °C. The prolonged duration of thermal oxidation significantly influences the morphological transformations and mechanical properties of the samples, surpassing the effects induced by exposure to a temperature of 110 °C. On the other hand, for sample SBC 818, the ductile fracture was observed after 20 days of oxidation at 90 °C.

**Figure 13:**
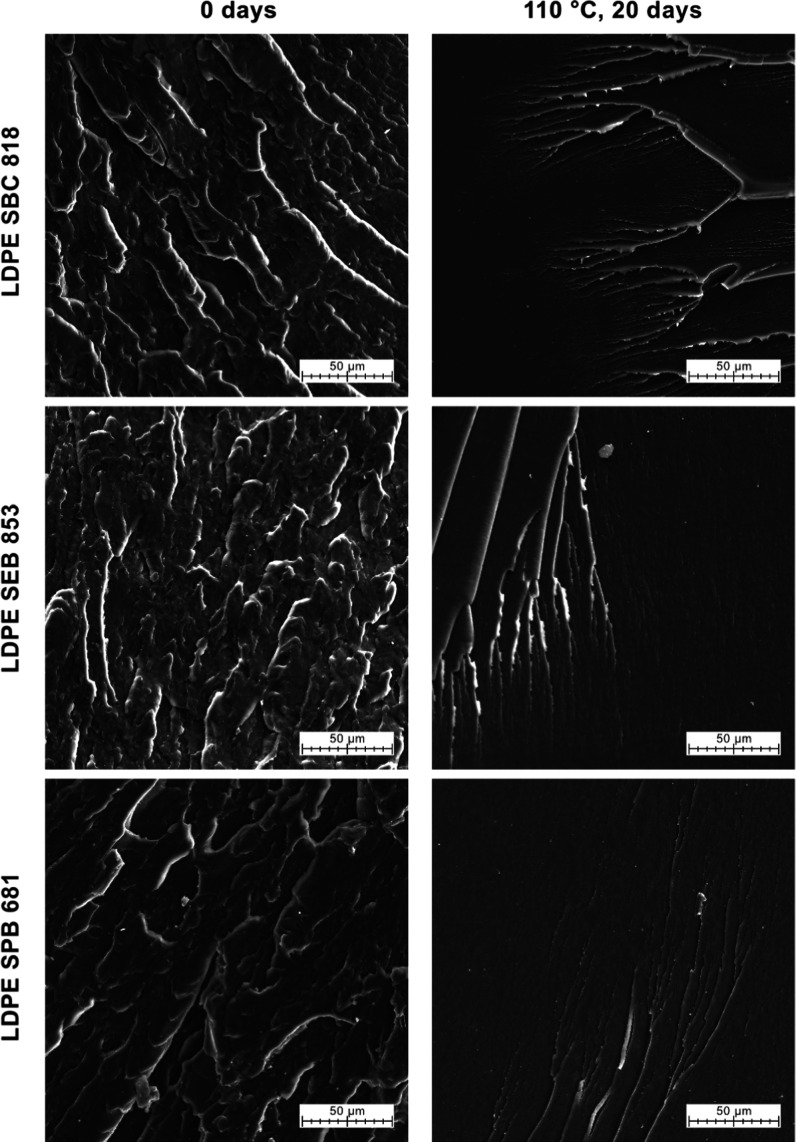
SEM images of the cross-section of unaged and thermally aged samples for 20 days at 110 °C.

## 5 Conclusions

The screening research focused on analyzing the impact of elevated temperatures (90–110 °C) in an extended time regime on the structure and properties of three varieties of biobased low-density polyethylene. Comprehensive spectroscopic, calorimetric, and rheological analyses allowed us to notice differences in the behavior of polymers depending on the dispersity and molecular weight. All assessed polyethylenes underwent the cross-linking process. However, the most significant tendency to this behavior was demonstrated by the most linear grade and characterized by the highest dispersity (SBC 818), while the earliest changes in the onset of degradative effects were noticed for the polymer with the lowest molecular weight and a high degree of branching (SEB 853). The observed decrease of crystallinity after thermal degradation was explained by the LDPE structures’ cross-linking. The primary conclusion from the analysis of the crystallinity changes determined by differential scanning calorimetry and X-ray diffractometry is that the dominant effect caused by the thermo-oxidative degradation is cross-linking, which surpasses the chain scission reaction for degradation at 110 °C. On the other hand, the analysis of the FTIR spectra suggests that degradation behavior is similar to the one caused by UV radiation described so far in the literature, where the carboxylic acid compound is dominant in carbonyl peak deconvolution. From rheological analysis, two trends in the course of the viscosity curves can be noted. The first is associated with a decrease in molecular weight due to degradation, resulting in a lower viscosity over the entire angular frequency range. The second is the disappearance of the complex viscosity curve transition to the first Newtonian range for samples subjected to long aging times, which is related to the formation of spatially cross-linked LDPE structures. This cross-linking effect co-occurred with the chain scission for samples subjected to thermooxidation at 100 and 110 °C. The decreasing degree of branching, dispersity, and increasing molecular weight of biobased polyethylene increase its tendency to form escaped structures. In addition, the aging temperature had a dominant role in changing the nature of the phenomena from degradation and chain scission to a progressive cross-linking process. The application conclusion from the conducted research, which contributes to the work carried out so far, is a comprehensive presentation of the impact of thermal effects on changes in biobased polyethylene structure. Dispelling doubts arising from using sustainable resources to synthesize polyethylene may significantly influence the development of nonbiodegradable thermoplastics of biological origin.

## Supplementary Material



## Data Availability

Data will be made freely available on request directed to the corresponding author.

## Notes

Distribution of the molecular weight of three raw grades of biobased LDPE (Figure S1); deconvoluted carbonyl bands after thermal oxidation for 20 days (Figure S2); scheme of appearance of nine distinguished carbonyl peaks (Figure S3); color of the samples presented as a parameter b and yellowness index (YI) (Figure S4); EDS mapping of oxygen in cross-section of aged samples in various temperature conditions (Figure S5–6); and SEM images of cryo-fractured samples (Figure S7–9) (PDF)
