# Supplementary material for: Unveiling the Effects of Thermal Aging on the Oxidative Stability of Biobased Low-Density Polyethylenes
Source: ACS Omega. 2025 Jun 11;10(24):25432–50. doi: 10.1021/acsomega.5c00636 (PMC12198993; doi:10.1021/acsomega.5c00636)
Supplement: Supplementary file 1 [file ao5c00636_si_001.pdf]

Unveiling the effects of thermal aging on the oxidative stability of bio-based low-density polyethylenes

Joanna Aniśko[1]**, *Anatolij Sokolohorskyj*[2], *Izabela Szafraniak-Wiza*[3], *Paulina Kosmela*[4], *Adam Piasecki*[3], *Mateusz Barczewski*[1]

[1] Poznan University of Technology, Faculty of Mechanical Engineering, Institute of Materials Technology, Polymer Processing Division Piotrowo 3, 61-138 Poznań, Poland
[2] University of Chemistry and Technology, Department of Polymers, Prague, Technická 5, 166 28 Prague 6, Czech Republic
[3] Poznan University of Technology, Faculty of Materials Engineering and Technical Physics, Institute of Materials Engineering, al. Jana Pawła II 24, 61-138 Poznań, Poland
[4] Gdansk University of Technology, Department of Polymer Technology, Narutowicza 11/12 80-233 Gdańsk, Poland
*corresponding author: joanna.anisko@put.poznan.pl

Supplementary Information

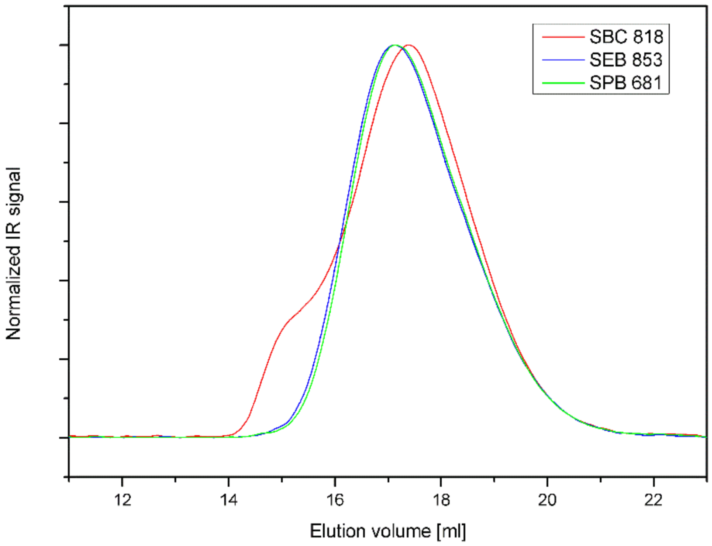

Fig. S1. Distribution of molecular weight of three raw grades of biobased LDPE.

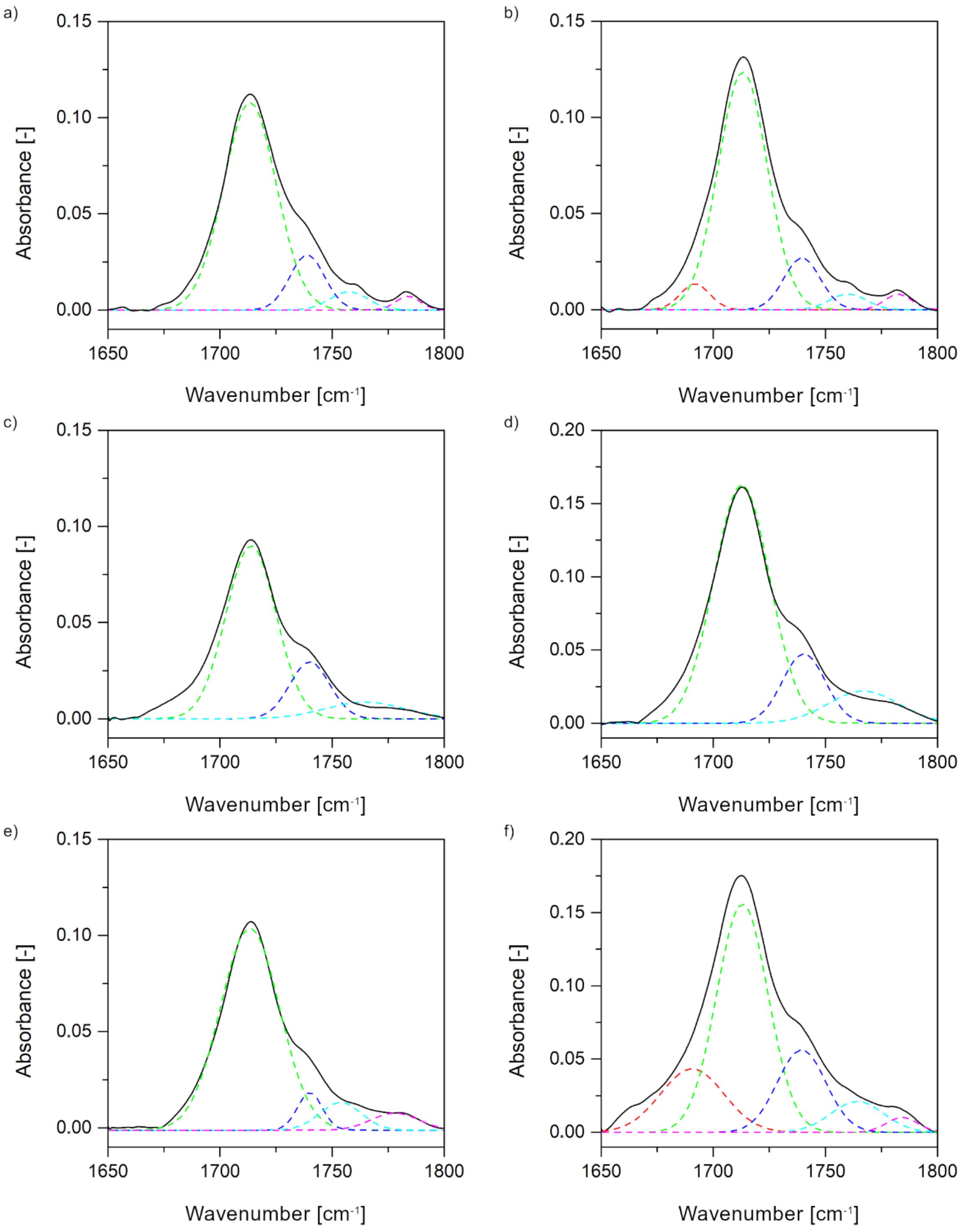

Fig. S2. Deconvoluted carbonyl bands after thermal oxidation for 20 days: LDPE SBC 818 90 °C (a) and 100 °C (b); LDPE SEB 853 90 °C (c) and 100 °C (d); LDPE SPB 681 90 °C (e) and 100 °C (f).

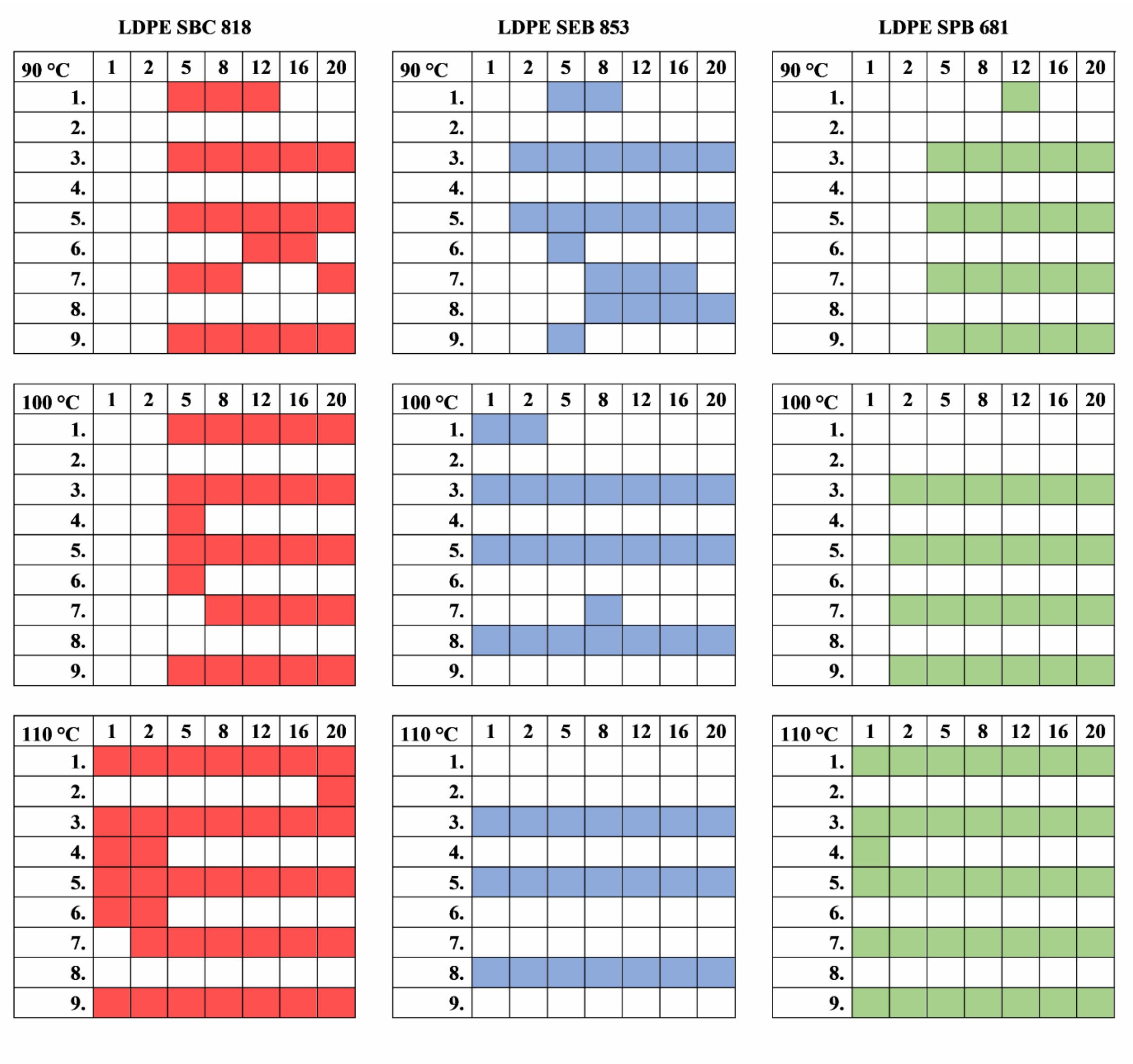

Fig. S3. The pattern of appearance of nine distinguished carbonyl peaks following temperature and time of thermal aging.

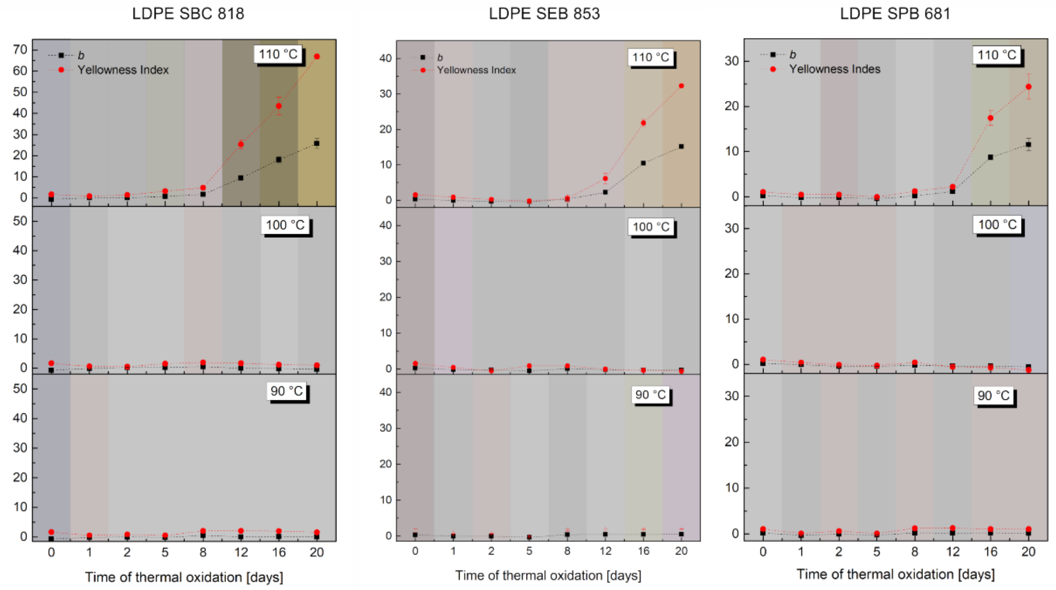

Fig. S4. Color of the samples presented as a parameter b and Yellowness Indes (YI).

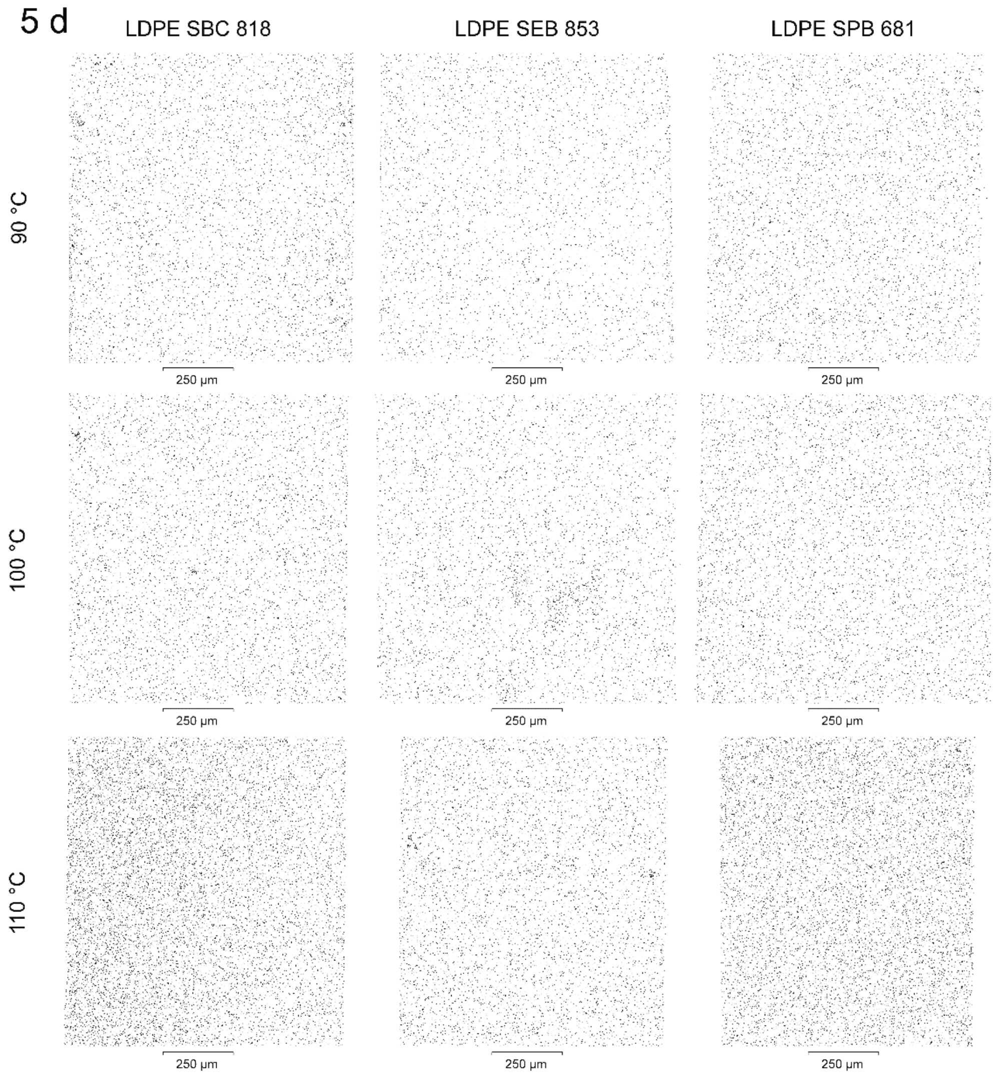

Fig. S5. EDS mapping of oxygen in cross-section of aged samples in various temperature conditions after 5 days; external surfaces left and right.

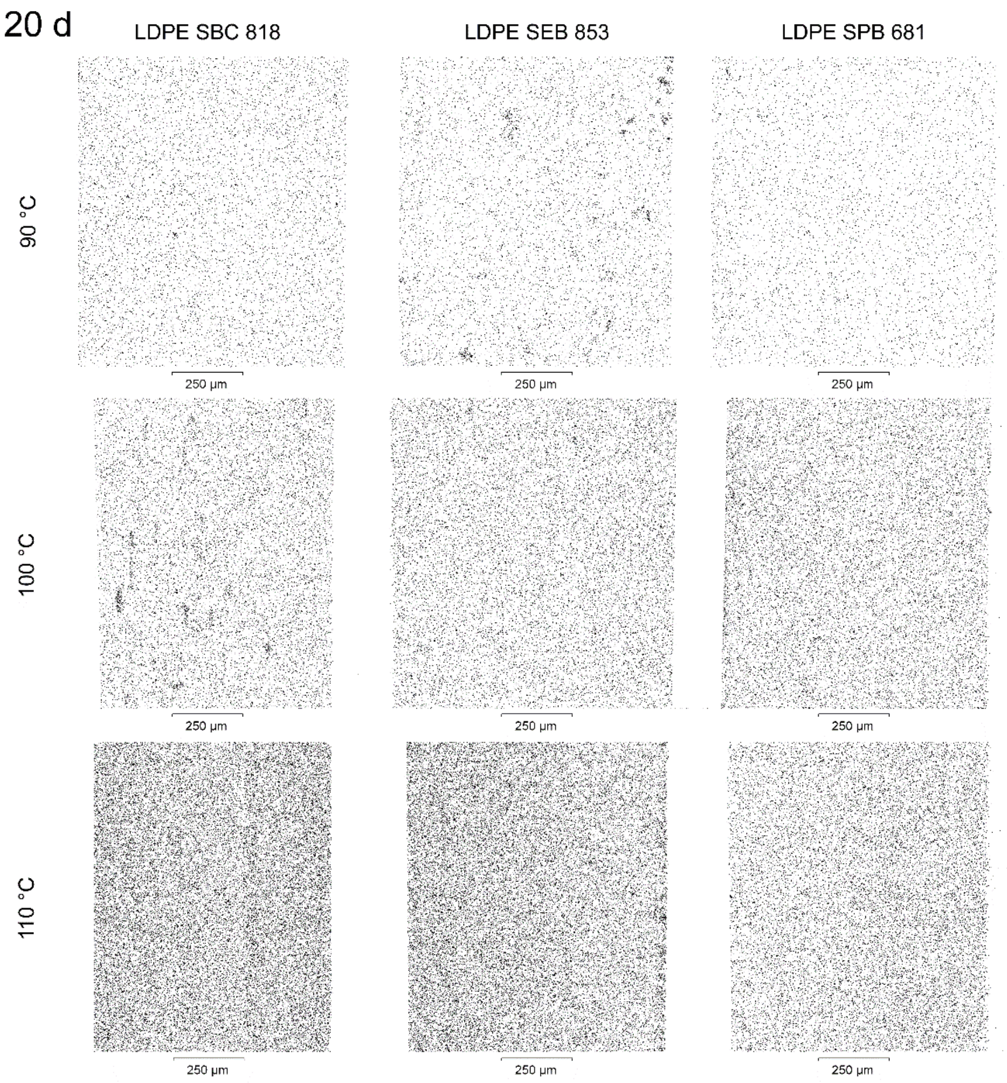

Fig. S6. EDS mapping of oxygen in cross-section of aged samples in various temperature conditions after 20 days; external surfaces left and right.

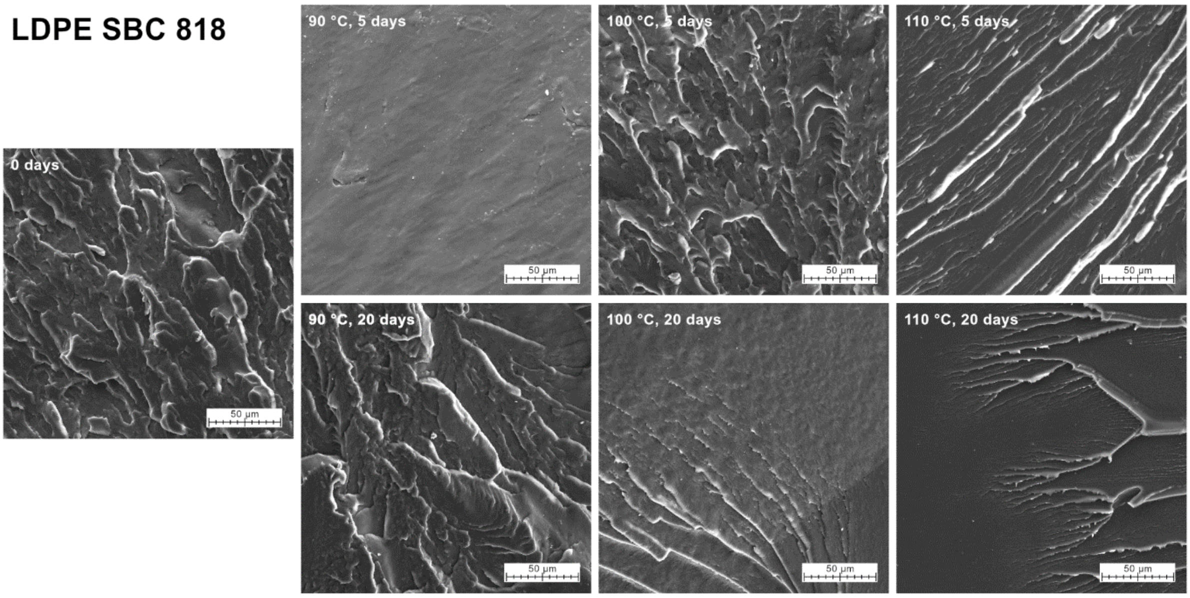

Fig. S7. Cross-section of thermally aged samples after 5 and 20 days of LDPE SBC 818.

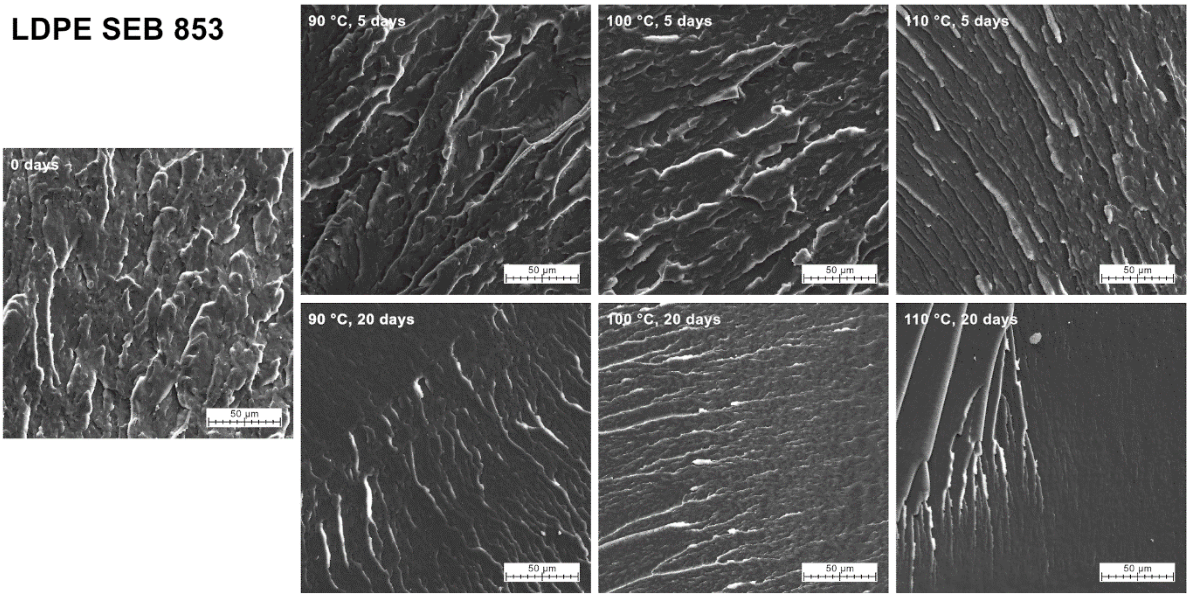

Fig. S8. Cross-section of thermally aged samples after 5 and 20 days of LDPE SEB 853.

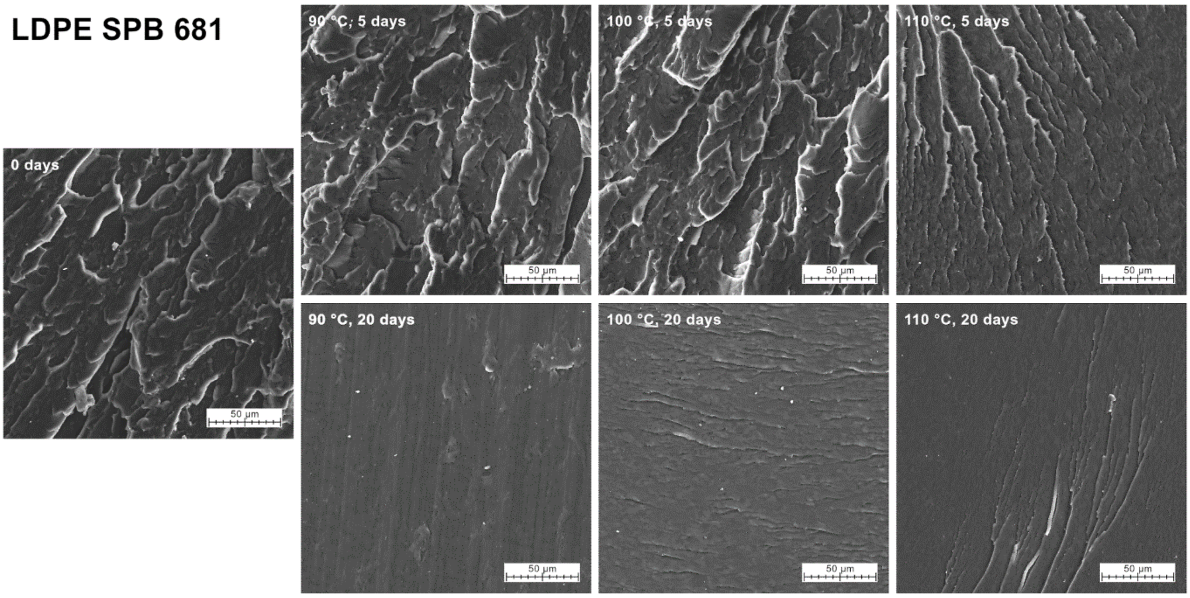

Fig. S9. Cross-section of thermally aged samples after 5 and 20 days of LDPE SPB 681.
